# Supplementary material for: Suppression of Choroidal Neovascularization by AAV-Based Dual-Acting Antiangiogenic Gene Therapy
Source: Mol Ther Nucleic Acids. 2019 Feb 2;16:38–50. doi: 10.1016/j.omtn.2019.01.012 (PMC6393707; doi:10.1016/j.omtn.2019.01.012)
Supplement: Document S1. Figures S1–S4 [file mmc1.pdf]

OMTN, Volume *16*

Supplemental Information

Suppression of Choroidal Neovascularization by AAV-Based Dual-Acting Antiangiogenic Gene Therapy

Anne Louise Askou, Sidsel Alsing, Josephine N.E. Benckendorff, Andreas Holmgaard, Jacob Giehm Mikkelsen, Lars Aagaard, Toke Bek, and Thomas J. Corydon

Figure S1

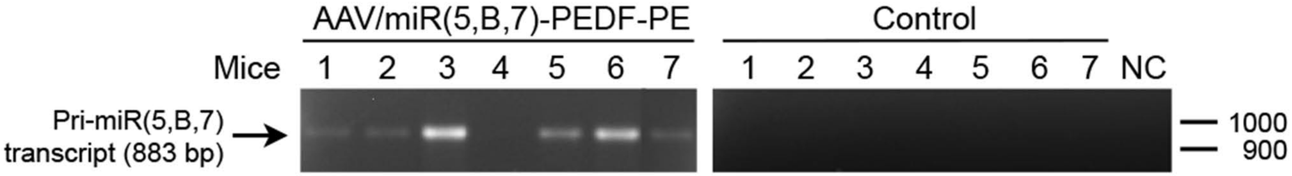
AAV/miR(5,B,7)-PEDF-PE
Control
Mice 1 2 3 4 5 6 7
1 2 3 4 5 6 7 NC
Pri-miR(5,B,7) transcript (883 bp)
1000
900

Figure S2

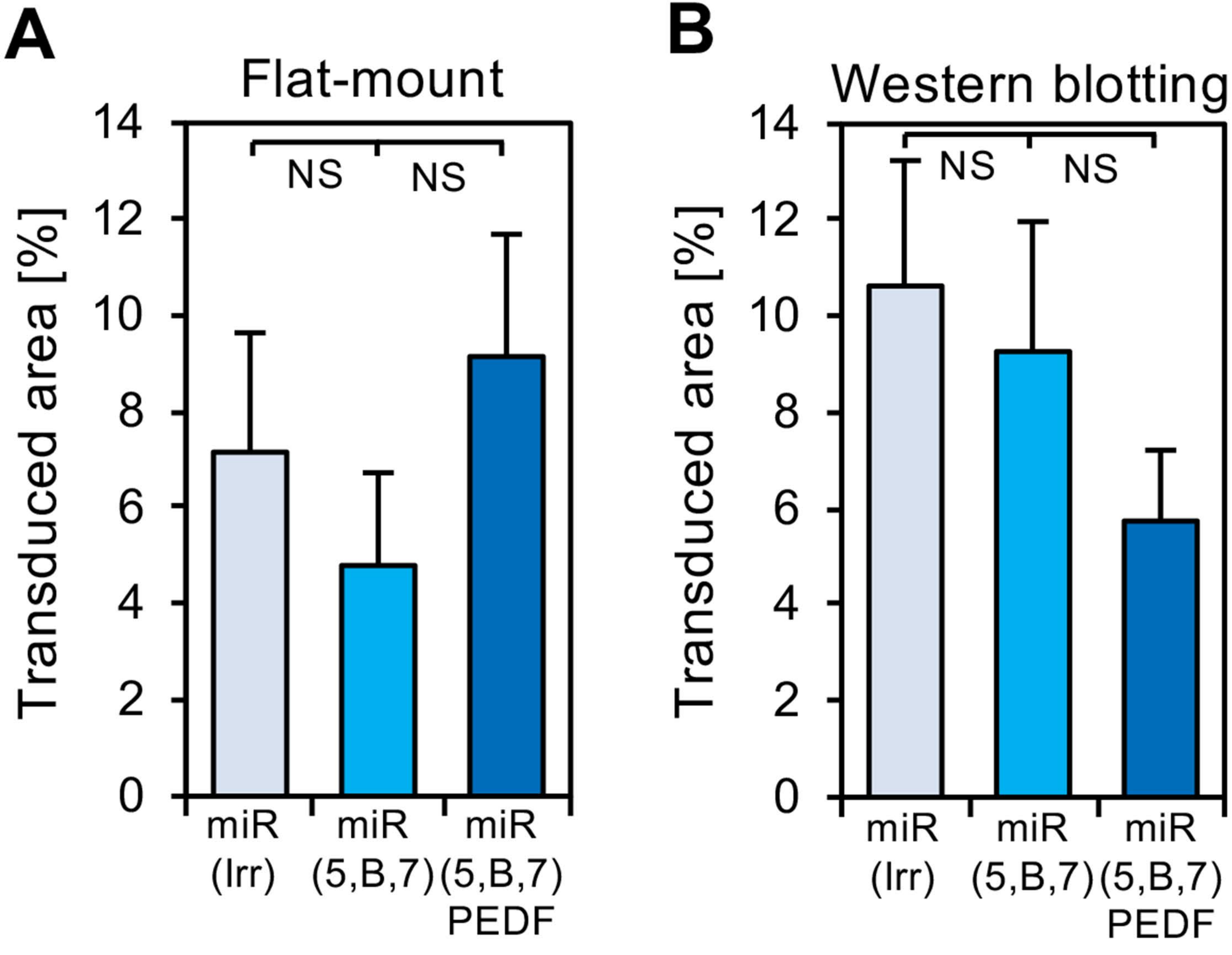
A
Flat-mount
Transduced area [%]
14
12
10
8
6
4
2
0
NS
NS
miR (Irr)
miR (5,B,7)
miR (5,B,7) PEDF
B
Western blotting
Transduced area [%]
14
12
10
8
6
4
2
0
NS
NS
miR (Irr)
miR (5,B,7)
miR (5,B,7) PEDF

Figure S3

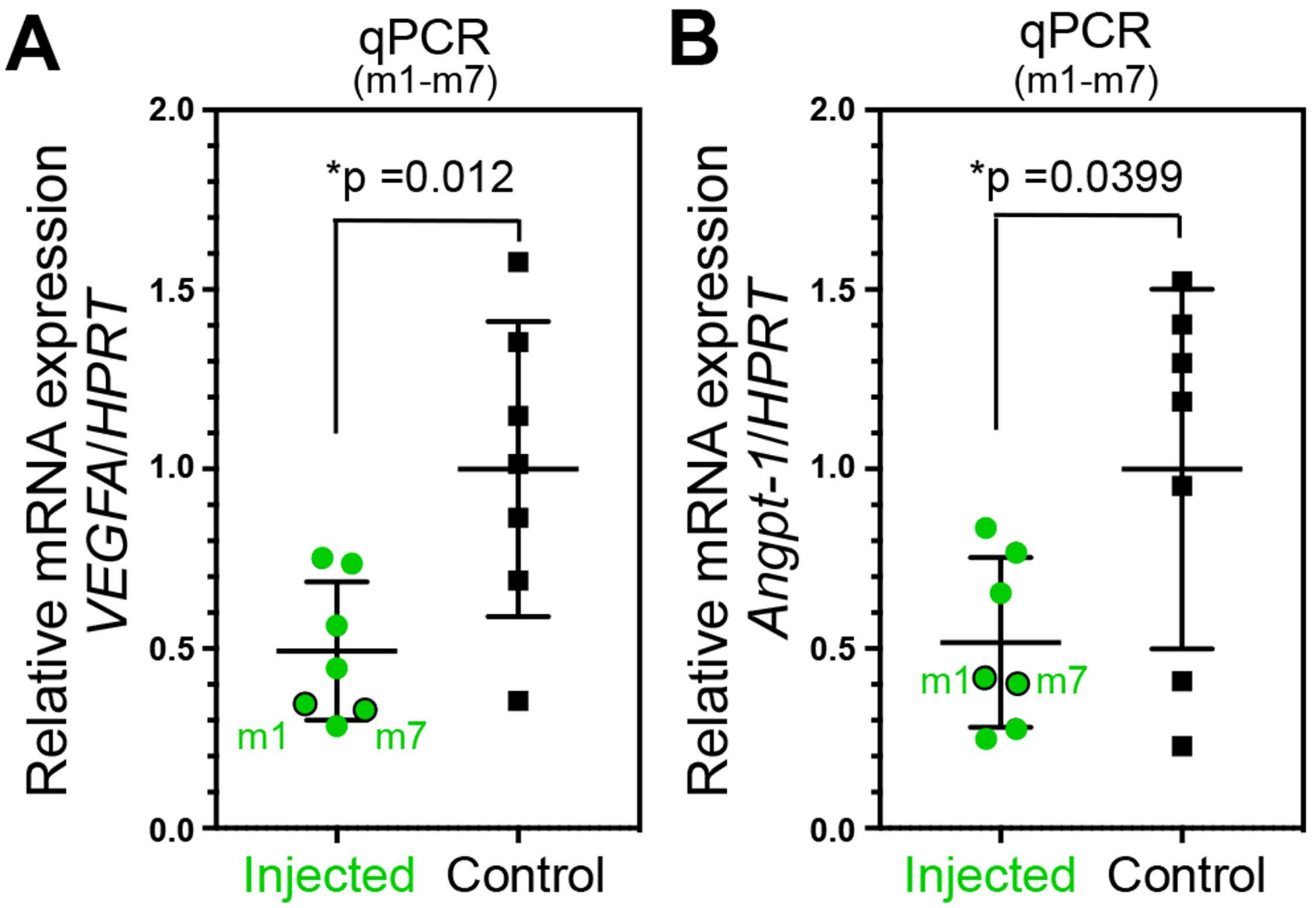
A
qPCR
(m1-m7)
2.0
1.5
1.0
0.5
0.0
Relative mRNA expression
VEGFA/HPRT
*p =0.012
m1
m7
Injected
Control
B
qPCR
(m1-m7)
2.0
1.5
1.0
0.5
0.0
Relative mRNA expression
Angpt-1/HPRT
*p =0.0399
m1
m7
Injected
Control

Figure S4

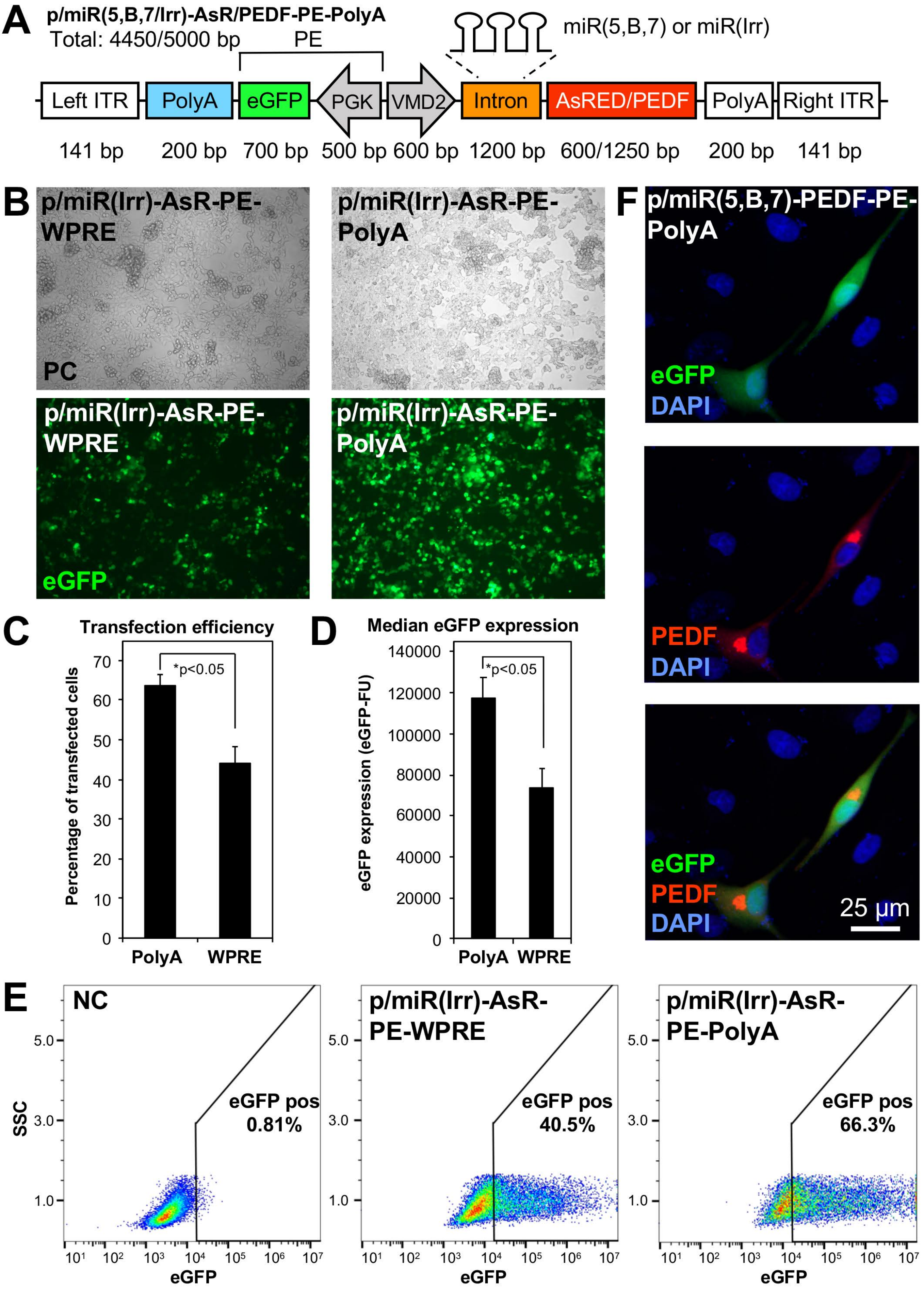

A
p/miR(5,B,7/Irr)-AsR/PEDF-PE-PolyA
Total: 4450/5000 bp
PE
miR(5,B,7) or miR(Irr)
Left ITR
PolyA
eGFP
PGK
VMD2
Intron
AsRED/PEDF
PolyA
Right ITR
141 bp
200 bp
700 bp
500 bp
600 bp
1200 bp
600/1250 bp
200 bp
141 bp
B
p/miR(Irr)-AsR-PE-WPRE
p/miR(Irr)-AsR-PE-PolyA
PC
p/miR(Irr)-AsR-PE-WPRE
p/miR(Irr)-AsR-PE-PolyA
eGFP
C
Transfection efficiency
Percentage of transfected cells
*p<0.05
PolyA
WPRE
D
Median eGFP expression
eGFP expression (eGFP-FU)
*p<0.05
PolyA
WPRE
E
NC
p/miR(Irr)-AsR-PE-WPRE
p/miR(Irr)-AsR-PE-PolyA
SSC
eGFP pos 0.81%
eGFP pos 40.5%
eGFP pos 66.3%
eGFP
F
p/miR(5,B,7)-PEDF-PE-PolyA
eGFP
DAPI
PEDF
DAPI
eGFP
PEDF
DAPI
25 µm

Figure S1 Expression of miRNA precursor transcript pri-miR(5,B,7) from AAV/miR(5,B,7)-PEDF-PE in the retina. Expression of the pri-miR(5,B,7) transcript from AAV/miR(5,B,7)-PEDF-PE in the retina was examined by RT-PCR. Twenty-eight dpi RPE cells were isolated from enucleated eyes and analyzed by PCR using cDNA as described in Materials and Methods. The contralateral, uninjected eye was used as control. A sample without cDNA was used as negative control (NC). Position of the pri-miR(5,B,7) transcript with the predicted size of 883 bp is indicated to the left. Positions of the 1000 and 900 bp bands of the molecular ruler are shown to the right. Bp, base pairs; NC, negative control.

Figure S2 Quantification of eGFP expression in the retina following delivery of AAV/miR(Irr/5,B,7)-AsRED 50 dpi. eGFP expression was assessed by fundoscopy in injected eyes included in the analysis shown in Figure 5 (n = 56). **A** Transduced area (percentage of total analyzed area) in the flat-mount group (n = 28). **B** Transduced area (percentage of total analyzed area) in the Western blotting group (n = 28). No statistically significant (indicted by NS) difference between the three groups was observed (one-way ANOVA followed by multiple comparisons (Tukey's multiple comparison test)), p-values in the range of 0.37-0.82 (flat-mount), and 0.34-0.92 (Western blotting). Error bars indicate SEM. Dpi, day post-injection; miR, microRNA; PEDF, pigment epithelium-derived factor.

Figure S3 Quantification of *VEGFA* and *Angpt-1* expression in RPE cells following subretinal delivery of AAV/miR(5,B,7)-PEDF-PE. In order to investigate the effect of reduced *VEGFA* levels on the angiogenic pathway *in vivo*, mRNA levels of the target *VEGFA* and of *Angpt-1* were assessed in RPE cells following a single subretinal injection (m1-m7). Twenty-eight dpi RPE cells were isolated from enucleated eyes and analyzed by RT-qPCR as described in Materials and Methods. Uninjected eyes were used as negative control. Mouse 1 and 7 indicated (green dots with black lines). **A** Assessment of the *VEGFA* expression (p = 0.012). **B** Assessment of the *Angpt-1* expression (p = 0.0399). The relative expression of *VEGFA* and *Angpt-1* in uninjected eyes were set to 1. *Statistically significant. Error bars indicate SEM. Angpt-1, Angiopoietin-1; dpi, day post-injection; miR, microRNA; PE, PGK-eGFP; PEDF, pigment epithelium-derived factor; VEGFA, Vascular endothelial growth factor A.

Figure S4 Optimization of the multigenic AAV vector. A Schematic presentation of the optimized p/miR(5,B,7/Irr)-AsR/PEDF-PE-PolyA vector. The WPRE sequence of the original vector presented in Figure 1 was replaced with the PolyA sequence. Numbers indicate size (in bp of the various components). The total sizes (in kb) of p/miR(5,B,7)-AsR/PEDF-PE-PolyA and p/miR(Irr)-AsR/PEDF-PE-PolyA are likewise indicated. **B** Visualization of eGFP expression in HEK-293 cells transfected with either p/miR(Irr)-AsR-PE-WPRE or p/miR(Irr)-AsR-PE-PolyA. Original magnification x10. **C-E** Flow cytometry analysis of HEK-293 cells transfected with either p/miR(Irr)-AsR-PE-WPRE or p/miR(Irr)-AsR-PE-PolyA. Untransfected cells were used as NC. **C-D** Assessment of the transfection efficiency and median eGFP expression following flow cytometry (mean ± SD). Students t-test, p = 0.006 (transfection efficiency) and p = 0.011 (expression). *Statistically significant. **E** Flow cytometry diagrams used to calculate the findings presented in C-D. The gaiting of the eGFP-positive cells is indicated. **F** Expression of eGFP (green) and PEDF in melanoma cells following transfection with p/miR(5,B,7)-PEDF-PE-PolyA. Visualization of PEDF was obtained following immunostaining using a mouse anti-PEDF-antibody. For visualization anti-PEDF antibodies were labeled with 568-ALEXA-anti-mouse antibodies (red). To visualize nuclei cells were stained with DAPI (blue). Scale bar = 25 µm. AsRED/AsR, red fluorescent marker protein; bp, base pairs; DAPI, 4',6-diamidino-2-phenylindol; eGFP, enhanced green fluorescent protein; FU, fluorescent unit; HEK-293, human embryonic kidney cells; H3, histone H3; Irr, irrelevant; ITR, inverted terminal repeat; kb, kilo bases; miR, microRNA; NC, negative control (untransfected cells); PE, PGK-eGFP; PEDF, pigment epithelium-derived factor; PC, phase contrast; PGK, phosphoglycerate kinase 1 promoter; PolyA, polyadenylation signal; SSC, side scatter; VMD2, vitelliform macular dystrophy 2 promoter.
