# Supplementary material for: Suppression of Choroidal Neovascularization by AAV-Based Dual-Acting Antiangiogenic Gene Therapy
Source: Mol Ther Nucleic Acids. 2019 Feb 2;16:38–50. doi: 10.1016/j.omtn.2019.01.012 (PMC6393707; doi:10.1016/j.omtn.2019.01.012)
Supplement: Document S2. Article plus Supplemental Information [file mmc2.pdf]

Molecular Therapy
Nucleic Acids
Original Article

Suppression of Choroidal Neovascularization by AAV-Based Dual-Acting Antiangiogenic Gene Therapy

Anne Louise Askou,[1] Sidsel Alsing,[1] Josephine N.E. Benckendorff,[1] Andreas Holmgaard,[1] Jacob Giehm Mikkelsen,[1] Lars Aagaard,[1] Toke Bek,[2] and Thomas J. Corydon[1,2]

[1]Department of Biomedicine, Aarhus University, 8000 Aarhus C, Denmark; [2]Department of Ophthalmology, Aarhus University Hospital, 8000 Aarhus C, Denmark

Vascular endothelial growth factor A (VEGFA) is involved in the pathogenesis of vasoproliferative retinal diseases, such as exudative age-related macular degeneration (AMD). The objective of this study was to investigate whether dual-acting therapy based on the simultaneous expression of anti-VEGFA microRNAs (miRNAs) and the secreted, antiangiogenic protein pigment endothelial-derived factor (PEDF) delivered by adeno-associated virus (AAV) vectors provides improved protection against choroidal neovascularization (CNV). To investigate this, a multigenic AAV vector allowing retina pigment epithelium (RPE)-specific expression of anti-VEGFA miRNAs and PEDF was engineered. Robust expression of PEDF, driven by the RPE-specific vitelliform macular dystrophy 2 promoter, was observed in human cells and in mouse retina. A significant reduction in CNV was observed in a laser-induced CNV mouse model 57 days post-injection of the AAV5 particles conveying either anti-VEGFA miRNA and PEDF dual therapy or anti-VEGFA miRNA monotherapy. Overall, CNV reduction was most prominent in animals receiving dual-acting therapy. In both cases, the reduction in CNV was accompanied by a significant attenuation of VEGFA. In conclusion, the presented data reveal that gene therapy targeting VEGFA via multigenic AAV vectors displays combined efficacy, suggesting that dual-acting therapy is an important tool in future eye gene therapy for the treatment of neovascular ocular diseases, including AMD.

INTRODUCTION

Adeno-associated virus (AAV)-based vectors are popular and well-accepted gene delivery vehicles for a wide variety of tissues,[1–6] and they represent promising tools for the treatment of retinal degenerations.[1–3,7–10] The non-pathogenic nature, long-term transgene expression in the absence of genome integration in both dividing and non-dividing cells, low immunogenicity, and ease of capsid swapping for the targeting of specific tissues are attractive features of recombinant AAV (rAAV) vectors.[11–13] These advances have made rAAV the vector of choice for most gene therapy applications to the eye, illustrated by several clinical trials[2,7,10,14] utilizing rAAV particles to ferry genes to specific retinal cells. Notably, Luxturna™, a gene therapy for patients with an inherited eye disease, was recently approved by the FDA.[15] The anatomical advantages, including shape, size, and accessibility of the eye, have accelerated research in gene therapy treatment of inherited retinal diseases and placed it at the forefront of gene-based technology development. Single-gene disorders are ideal candidates for gene supplementation or replacement, which is best suited for loss-of-function mutations, such as those involved in Leber's congenital amaurosis type 2 (*RPE65*),[1–3] retinitis pigmentosa (*MERTK*),[7,8] and choroideremia (*CHM*).[9,10]

In all of these trials, a normal gene was delivered via rAAV vectors to the retinal cells missing the healthy gene product. Unfortunately, the packaging capacity of the rAAV vector (<5 kb) is a limitation for the use of this vector system to treat diseases affecting the retina, which often requires the transfer of sequences that are longer than 5 kb.[16,17] Other viral vectors, such as lentiviral vectors (LVs), are needed to carry the large coding sequences. However, LV-based systems have certain intrinsic disadvantages, limiting their use for *in vivo* retinal gene therapy. Due mainly to the large size of the LV particles, transduction is limited to retina pigment epithelium (RPE) cells following a subretinal injection.[18] Moreover, LVs carry the risk of genotoxicity caused by insertional mutagenesis.[19] Another approach for treating diseases requiring transfer of a sequence larger than 5 kb is to exploit rAAV split-vector systems, where the coding sequence of a large protein has been split between two or more vectors, thereby increasing transfer capacity up to 9 kb for the dual-vector system[20,21] and up to 14 kb for triple vectors.[22,23]

Gene therapy has also been applied to acquired retinal diseases, such as neovascular age-related macular degeneration (nAMD) (ClinicalTrails.gov: NCT00109499, NCT01494805, NCT01024998, NCT01301443, NCT00363714, NCT00713518). nAMD is the leading cause of blindness in the western world, and the disease is currently treated by repetitive, often monthly intraocular injections of anti-vascular endothelial growth factor (VEGF) drugs (e.g., antibodies or traps) to maintain vision.[24–26] However, nAMD is a complex and

Received 21 June 2018; accepted 28 January 2019;
https://doi.org/10.1016/j.omtn.2019.01.012.

Correspondence: Thomas J. Corydon, Department of Biomedicine, Aarhus University, Wilhelm Meyers Allé 4, 8000 Aarhus C, Denmark.
E-mail: corydon@biomed.au.dk

 © 2019 The Author(s).
This is an open access article under the CC BY license (http://creativecommons.org/licenses/by/4.0/).

multifactorial disease caused by multiple genetic and environmental factors, and it is characterized by progressive degeneration of the outer retinal layers.[27,28] This stimulates neovascularization from the choroid into the sub-RPE space and the retina to disrupt the normal retinal anatomy.

The advent of anti-VEGF therapy more than a decade ago dramatically changed the treatment modality for nAMD patients, but anti-VEGF as a monotherapy is reaching its limits.[29,30] The current landscape in new treatment concepts for nAMD and other neovascular retinal diseases suggests that combination therapy, i.e., delivery of two or more therapeutics, may soon become reality, as indicated by multiple clinical trials combining two drugs for the treatment of neovascular nAMD, all with study start dates in 2017 (ClinicalTrials.gov: NCT03211234, NCT03034772, NCT03345082, NCT02806752, NCT03022318).

Notably, due to the involvement of multiple dysregulated pathways, each playing a significant role in the pathogenesis of AMD,[31,32] attention has been drawn to the development of combined therapies either targeting angiogenesis or other involved pathways. Hence, recent studies have investigated the efficacy of combination therapy. In a clinical trial, Nguyen and co-workers[33] found the combination of a small interfering RNA (siRNA) designed to target *VEGF* and ranibizumab to be efficacious, even though repeated injections of the dual-target therapy were still required. To take the concept of combinational treatment a step further, we have recently developed a multigenic LV, enabling the simultaneous expression of multiple anti-VEGFA microRNAs (miRNAs) and fluorescent reporter genes for the visualization of efficient cell transduction and effective production of antiangiogenic miRNAs in target cells.[34,35] Cell-specific, robust, and stable expression was obtained in RPE cells for up to 9 months following a single injection of LVs encoding therapeutic anti-VEGFA miRNAs expressed from the RPE-specific vitelliform macular dystrophy 2 (VMD2) promoter. Remarkably, significant *VEGFA* silencing resulted in reduced choroidal neovascularization (CNV) size in the laser-induced CNV mouse model following subretinal delivery of the multigenic vector,[36] suggesting that virus-based gene delivery is a viable option for sustained, combinational treatment of retinal neovascular diseases.

In the multigenic vector, expression of antiangiogenic miRNAs can be combined with the delivery of therapeutic proteins, such as antiangiogenic factors for retinal support.[34] Pigment endothelial-derived factor (PEDF), a widely expressed multifunctional member of the serine proteinase inhibitor (serpin) family,[37] is one such protein.[38] Several studies have pinpointed PEDF as a critical player in many physiological and pathophysiological processes, including neuroprotection, angiogenesis, and inflammation.[38–42] Interestingly, unbalanced vitreous levels of PEDF were found in patients with diabetic retinopathy,[43,44] and reduced levels of PEDF have been found in patients with CNV due to AMD.[45] Thus, co-delivery of PEDF within angiogenic sites could be a promising strategy for the treatment of angiogenesis-related diseases.

This study aimed to investigate the *in vivo* antiangiogenic effect of multigenic AAV vectors encoding PEDF as well as multiple miRNAs targeting the *VEGFA* gene. AAV5 particles delivered by subretinal injections provided widespread and RPE-specific expression in the murine retina. A significant reduction in CNV due to *VEGFA* knockdown was observed in the laser-induced CNV mouse model following the administration of AAV5 particles encoding antiangiogenic molecules. This study is the first attempt to test the multigenic system in AAV vectors, and the provided data suggest that dual-acting antiangiogenic gene therapy based on multigenic AAV vectors is an important therapeutic tool in future eye gene therapy for CNV-related diseases.

RESULTS

Development of Multigenic AAV Vectors

Multigenic LVs encoding antiangiogenic miRNAs (miR5,B,7) and proteins have been engineered and thoroughly described in our previous studies.[34–36] Specifically, correct processing of the three miRNAs (miR5, miRB, and miR7) from the intron region of our expression cassette, resulting in ~21- to 23-nt-long mature miRNAs, was verified by northern blotting.[34] Notably, similar amounts of miRNA were processed from miRNA clusters containing one relevant (miR5, miRB, or miR7) and two irrelevant miRNAs (miRS1–S3), e.g., miR(5,S2,S3), miR(S1,B,S3), or miR(S1,S2,7), compared to the cluster harboring all three miRNAs, miR(5,B,7). This indicates that miRNA processing was not affected by the sequence of adjacent miRNAs in the cluster.[34] These vectors both harbor the VMD2 promoter[34,46] for robust RPE-specific expression of antiangiogenic molecules and EGFP driven by the phosphoglycerate kinase 1 (PGK) promoter. The expression cassettes encode three miRNAs and AsRED (AsR, a red fluorescent protein) or PEDF, transcribed as a single mRNA by the VMD2 promoter, and EGFP driven by the PGK promoter (Figure 1A). The polycistronic miRNA cluster is intron embedded, and, upon processing, all three miRNAs (miR5, miRB, and miR7) were designed to perfectly target *mVEGFA* mRNA.[34] For the production of AAV5 particles, the two back-to-back RNA polymerase II (Pol II)-driven expression cassettes were cloned into the pAAV/siRNA shuttle vector, resulting in the three plasmids entitled p/miR(Irr)-AsR-PE, p/miR(5,B,7)-AsR-PE, and p/miR(5,B,7)-PEDF-PE (Figure 1A).

To validate RPE-specific expression of fluorescence markers from the new AAV vectors, melanoma cells, which enable transcription from VMD2-driven expression cassettes *in vitro*,[34] were transfected with p/miR(Irr)-AsR-PE, p/miR(5,B,7)-AsR-PE, and p/miR(5,B,7)-PEDF-PE (Figure 1B). Green fluorescence was detected in melanoma cells transfected with each of the three constructs, and red fluorescence was detected from melanoma cells transfected with p/miR(Irr)-AsR-PE and p/miR(5,B,7)-AsR-PE, indicating full functionality of the constructs. Moreover, PEDF expression was investigated by western blot analysis (Figure 1C) and fluorescence microscopy (Figure 1D). Both methods verified vector-encoded expression of PEDF in transfected melanoma cells, and, furthermore, western blot analysis revealed secretion of a 46-kDa protein into the medium, consistent with the size of PEDF. Together with previous studies,[34,36] these

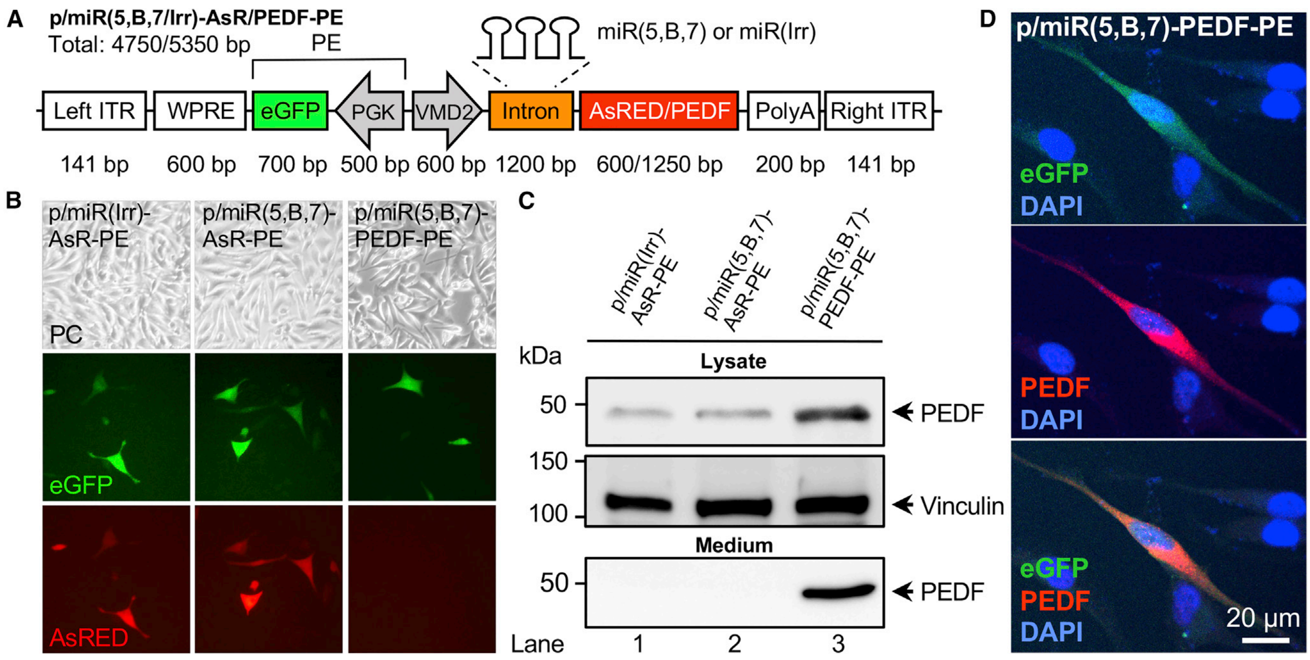

Figure 1. Design and Validation of p/miR(5,B,7/Irr)-AsR/PEDF-PE Vectors *In Vitro*

(A) Schematic presentation of the AAV-based p/miR(5,B,7/Irr)-AsR/PEDF-PE multigenic expression vector. The AAV vector contains the RPE-specific VMD2 promoter, which simultaneously drives the expression of a microRNA cluster, either miR(5,B,7) targeting *VEGFA* or miR(Irr) (irrelevant control miRNAs) and the fluorescent marker protein AsRED (red). In a later version of the vector, the AsRED sequence was replaced by the cDNA sequence encoding the antiangiogenic protein PEDF. Furthermore, in a back-to-back orientation, the vector contains the fluorescent marker protein EGFP (green) expressed from the ubiquitously active PGK promoter. Numbers indicate size (in bp of the various components). The total sizes (in kb) of p/miR(5,B,7)-AsR/PEDF-PE and p/miR(Irr)-AsR-PE are likewise indicated. (B) Assessment of EGFP and AsRED expression following transient transfection in melanoma cells. Original magnification × 10. (C) Western blotting of samples from melanoma cells transfected with p/miR(Irr)-AsR-PE, p/miR(5,B,7)-AsR-PE, or p/miR(5,B,7)-PEDF-PE plasmids. The cell lysate (40 µg total protein per lane) and medium fractions were subjected to electrophoresis, blotting, and immunostaining using a mouse anti-PEDF antibody. Molecular weight marker is shown to the left. Positions of PEDF and the vinculin loading control are indicated with arrowheads to the right. (D) Expression of EGFP (green) and PEDF in melanoma cells transfected with p/miR(5,B,7)-PEDF-PE. The expression of PEDF was validated following immunostaining using a mouse anti-PEDF antibody. For visualization, anti-PEDF antibodies were labeled with 568 Alexa Fluor goat-anti-mouse (red). Cells transfected with empty vector showed no signal (data not shown). To visualize nuclei, cells were stained with DAPI (blue). Scale bar, 20 µm. AsRED (AsR), red fluorescent marker protein; DAPI, 4′,6-diamidino-2-phenylindol; ITR, inverted terminal repeat; PE, PGK-EGFP; PEDF, pigment epithelium-derived factor; PC, phase contrast; PGK, phosphoglycerate kinase 1 promoter; poly(A), polyadenylation signal; VMD2, vitelliform macular dystrophy 2 promoter; WPRE, woodchuck hepatitis virus posttranscriptional regulatory element.

observations verified functionality of the expression cassettes, and vectors were packaged in AAV5 capsids.

PEDF Expression in the Murine Retina

The *PEDF* expression from the AAV vectors was examined by unilateral, subretinal injection of 4.2×10^9 vector genomes (vg) AAV/miR(5,B,7)-PEDF-PE in 15 mice (m1–m7 and m9–m16). Transduction of retinal cells was assessed by fluorescence fundoscopy (Figure 2A) 28 days post-injection (dpi), showing robust expression of EGFP in 13 of 16 injected animals. Expression of *PEDF* from the AAV vectors in the retina was examined by real-time qPCR of *PEDF* mRNA (Figure 2B) isolated from RPE cells and by western blot analysis of PEDF protein levels in the neuroretina (Figures 2C and 2D). The uninjected contralateral eye from the same mice were used as controls.

PEDF mRNA (m1–m7) and PEDF protein (m9–m16) levels were significantly increased ($p = 0.0021$, qPCR; and $p = 0.0009$, western blotting) in all analysed eyes. The increase correlated with the size of the transduced area, i.e., a larger increase in eyes with a larger transduced area (see m1, m7, m12, and m15 in Figure 2). Expression of the miRNA precursor transcript pri-miRNA(5,B,7) from AAV/miR(5,B,7)-PEDF-PE in the RPE cells was examined by RT-PCR using cDNA obtained from the isolated mRNA. As shown in Figure S1 a pri-miRNA(5,B,7)-specific amplicon of 883 bp was detected in 6 of 7 mice. The fact that the pri-miRNA(5,B,7) transcript was not detected in mouse 4 correlated with the low amount of *PEDF* mRNA revealed by qPCR (Figure 2B) and a limited transduced area (data not shown). Together, these findings demonstrate that the pri-miRNA(5,B,7) cluster and PEDF were co-expressed *in vivo*.

Multigenic AAV Vectors Efficiently Transduce RPE Cells following Subretinal Delivery in Mice

Verification of functional expression *in vivo* was assessed in the murine retina following a single subretinal injection of 4.2×10^9 vg AAV/miR(Irr)-AsR-PE, AAV/miR(5,B,7)-AsR-PE, or AAV/miR

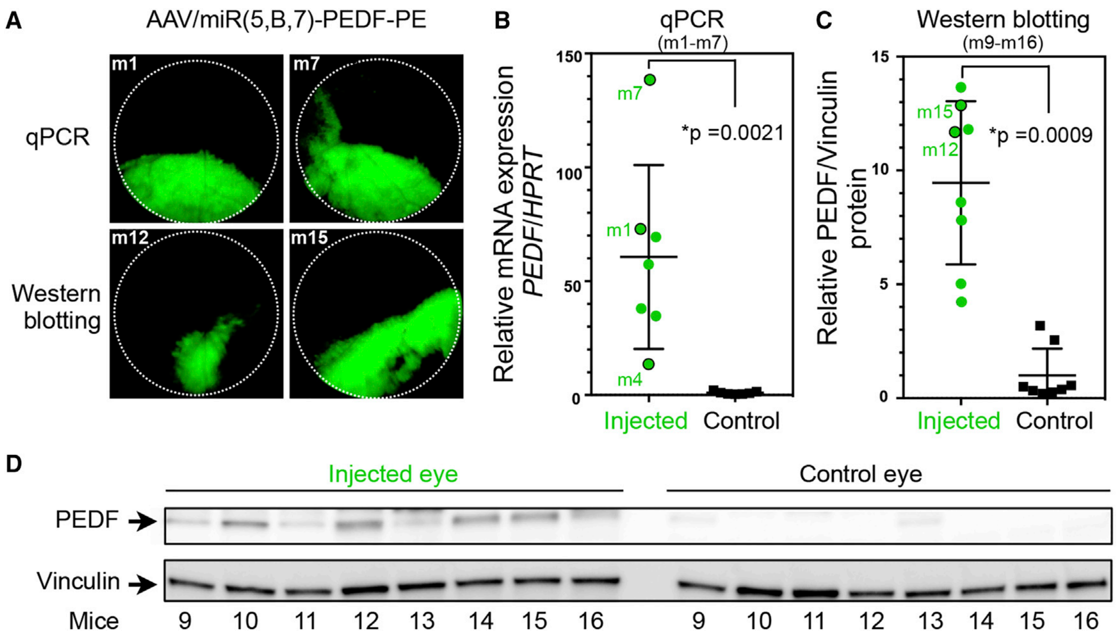

Figure 2. *In Vivo* Expression of PEDF following Subretinal Administration of AAV/miR(5,B,7)-PEDF-PE, Assessed 28 dpi

(A) Fundus images of the retina from representative mice (m1, m7, m12, and m15, likewise indicated in B–D) 28 days after subretinal injection of AAV/miR(5,B,7)-PEDF-PE. Eyes isolated from m1–m7 (n = 7) were used for real-time qPCR (shown in B), whereas eyes derived from m9–m16 (n = 8) were used for western blot analysis (depicted in C and D). (B) Assessment of *PEDF* mRNA expression in RPE cells isolated from 7 mice (m1–m7). In each mouse, one eye was injected with AAV vectors (4.2×10^9 vg) whereas the contralateral, uninjected eye served as control (Control). At 28 dpi, RNA was purified from the RPE cells and real-time qPCR was conducted. The *PEDF* expression was related to the expression of the *HPRT* housekeeping gene. The expression of *PEDF* in the uninjected eye was set to 1. Mice 1, 4 and 7 are indicated (green dots with black lines). (C) Semi-quantification of the PEDF protein amount expressed in retinal cells (mice m9–m16). Total protein was obtained from neuroretina isolated from AAV-treated (4.2×10^9 vg AAV/miR(5,B,7)-PEDF-PE) and uninjected eyes. A total of 15 μg protein was loaded in each lane. Following electrophoresis and western blotting, the PEDF level was visualized with a mouse-anti-PEDF antibody. Antibodies against vinculin were utilized for the loading control. The amount of PEDF relative to vinculin was quantified as described in the Materials and Methods. The relative expression of PEDF in the uninjected eye was set to 1. Mice 12 and 15 are indicated (green dots with black lines). (D) Images of western blots used for the quantification shown in (B). Positions of PEDF and the vinculin loading control are indicated with arrowheads to the left. Mice 9–16 are indicated below the blots. For AAV/miR(5,B,7)-PEDF-PE versus uninjected, p values were p = 0.0021 (qPCR) and p = 0.0009 (western blotting). *Statistically significant. Error bars indicate SEM. HPRT, hypoxanthine phosphoribosyltransferase; PE, PGK-EGFP; PEDF, pigment epithelium-derived factor; vg, vector genomes.

(5,B,7)-PEDF-PE. Transduction efficiency of the vectors was assessed 50 dpi via fluorescence fundoscopy (Figure 3), which showed similar transduction efficiency among the three different vectors. Noticeably, the size of the transduced area differed among mice in each group. This observed difference was most likely due to variations in injection site and angle, since mice in each group were injected on the same day with virus from the same preparation. Quantification of the EGFP signal (transduced area) in the injected animals used for CNV size assessment on flat-mounts and western blotting analysis showed no statistically significant difference (p values in the range of 0.37–0.82 [flat-mount] and 0.34–0.92 [western blotting]) among the three groups (Figure S2).

Ex vivo fluorescence microscopy of RPE and choroidal flat-mounts 57 dpi revealed the efficient transduction of RPE cells in mice injected with AAV/miR(Irr)-AsR-PE and AAV/miR(5,B,7)-AsR-PE, as judged by EGFP expression (Figures 4A and 4D) and AsRED expression (Figures 4B and 4E). In contrast, EGFP expression in RPE cells in mice injected with AAV/miR(5,B,7)-PEDF-PE (Figure 4G) was markedly lower. As expected, no AsRED expression was detected in animals injected with AAV/miR(5,B,7)-PEDF-PE (Figure 4H). Furthermore, the *ex vivo* fluorescence microscopy of the RPE and choroidal flat-mount clearly showed that the antiangiogenic expression cassette was active (AsRED-positive cells) in all of the transduced cells (EGFP-positive cells; Figures 4B, 4C, 4E, and 4F).

CNV Reduction by the Delivery of Multigenic AAV Vectors Expressing Antiangiogenic Molecules

Investigation of the antiangiogenic effect of the multigenic AAV vectors was carried out by subretinal administration of 4.2×10^9 vg AAV/miR(Irr)-AsR-PE, AAV/miR(5,B,7)-AsR-PE, or AAV/miR(5,B,7)-PEDF-PE. At day 0, three groups of 20 mice were unilaterally injected (Figure 5A). At day 50, *in vivo* fundoscopy revealed efficient, albeit variable, transduction in the retina in all three groups (Figure 3). For analysis of CNV size, four laser burns were applied in each injected eye outside the transduced area. 7 days after the aforementioned laser rupture of Bruch's membrane, mice were sacrificed, eyes were enucleated and dissected, and flat-mounts were stained

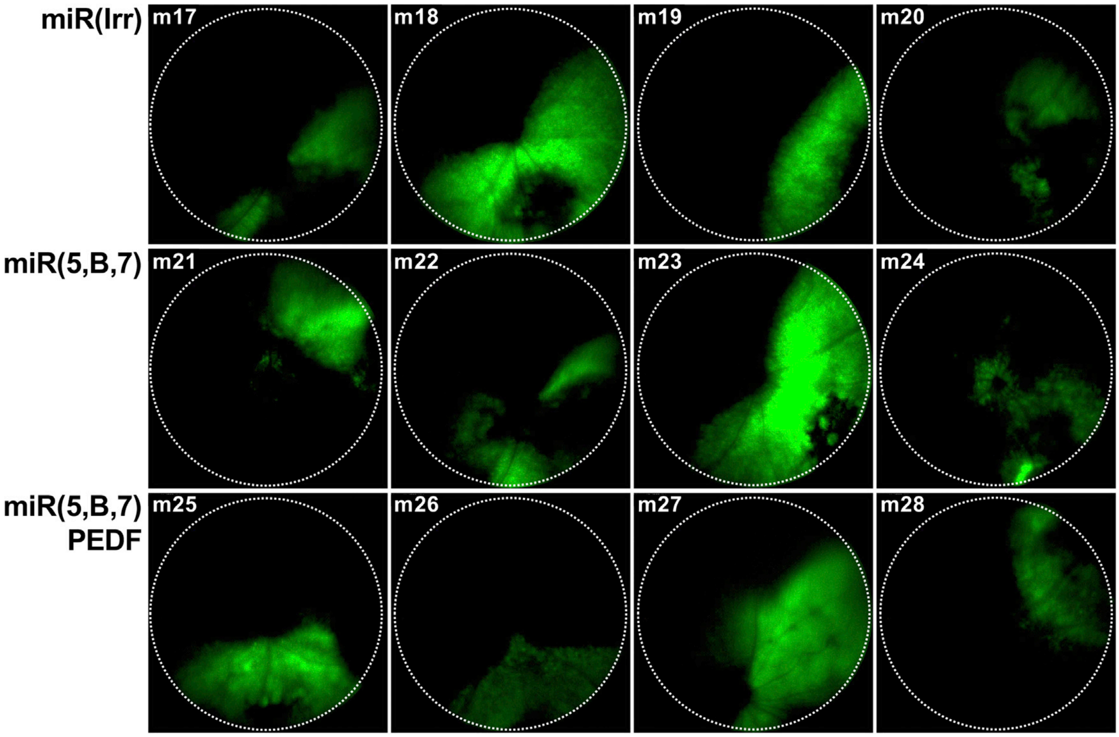

Figure 3. ***In Vivo*** **Expression of EGFP Co-expressed with miR(Irr/5,B,7)-AsR, Assessed by Fundoscopy 50 dpi**
Representative fundus images of the retina from individual mice following subretinal injection of 4.2 $\times$ 10^9 vg AAV/miR(Irr)-AsR-PE (m17-m20), AAV/miR(5,B,7)-AsR-PE (m21-m24), and AAV/miR(5,B,7)-PEDF-PE (m25-m28), respectively. Dotted, circular lines indicate the area analyzed for EGFP expression shown in Figure S2. AsRED (AsR), red fluorescent marker protein; vg, vector genomes.

with isolectin (Figures 5A and 5B). CNV area measurements revealed a statistically significant reduction in CNV area of approximately 34% and 45% in mice injected with AAV/miR(5,B,7)-AsR-PE and AAV/miR(5,B,7)-PEDF-PE, respectively, compared to animals receiving AAV/miR(Irr)-AsR-PE (Figure 5C). Interestingly, the CNV reduction was improved, however, not to statistical significance, in animals receiving combinational antiangiogenic therapy compared to animals treated only with anti-VEGFA miRNAs.

To verify that the detected reduction in CNV area coincided with the suppression of VEGFA, western blotting was performed on whole-eye cellular extracts obtained from mice likewise injected with 4.2 $\times$ 10^9 vg AAV/miR(Irr)-AsR-PE, AAV/miR(5,B,7)-AsR-PE, and AAV/miR(5,B,7)-PEDF-PE. To verify changes in VEGF expression, ten laser burns were applied in each injected eye outside the transduced area.[36,47–49] 3 days after laser rupture of Bruch's membrane, mice were sacrificed and western blotting was performed (Figure 5D). Quantification of VEGFA levels showed a reduction of 32% in mice injected with AAV/miR(5,B,7)-AsR-PE compared to controls injected with AAV/miR(Irr)-AsR-PE (Figure 5E). Notably, mice injected with AAV/miR(5,B,7)-PEDF-PE showed a significant reduction in VEGFA levels of 65% compared to controls (Figure 5E). These findings advocate that reduced VEGFA levels as a consequence of AAV-delivered antiangiogenic molecules caused the observed reduction in CNV.

To support the evidence of antiangiogenic effect in general, mRNA levels of the target *VEGFA* and angiopoietin-1 (*Angpt-1*), a player in the angiogenic pathway,[50,51] were analyzed in mice injected with 4.2 $\times$ 10^9 vg AAV/miR(5,B,7)-PEDF-PE. 28 days after injection, RPE cells were isolated and total RNA was purified. Following cDNA synthesis, the *VEGFA* and *Angpt-1* expression levels were analyzed by real-time qPCR. As shown in Figure S3A, the *VEGFA* expression was significantly reduced in eyes injected with AAV/miR(5,B,7)-PEDF-PE compared to uninjected control eyes ($p = 0.012$). Notably, the level of *Angpt-1* mRNA was likewise significantly reduced ($p = 0.0399$) in injected animals compared to control eyes (Figure S3B). In support of the findings presented in Figure 2, mice with the lowest expression of *VEGFA* and *Angpt-1* (mice 1 and 7) also displayed high expression of PEDF and EGFP.

DISCUSSION

In previous studies,[34–36] we have investigated the expression from and efficacy of lentivirus-based vectors containing the polycistronic miRNA cluster, and, most recently, we demonstrated the suppression of CNV in a CNV mouse model following a single subretinal

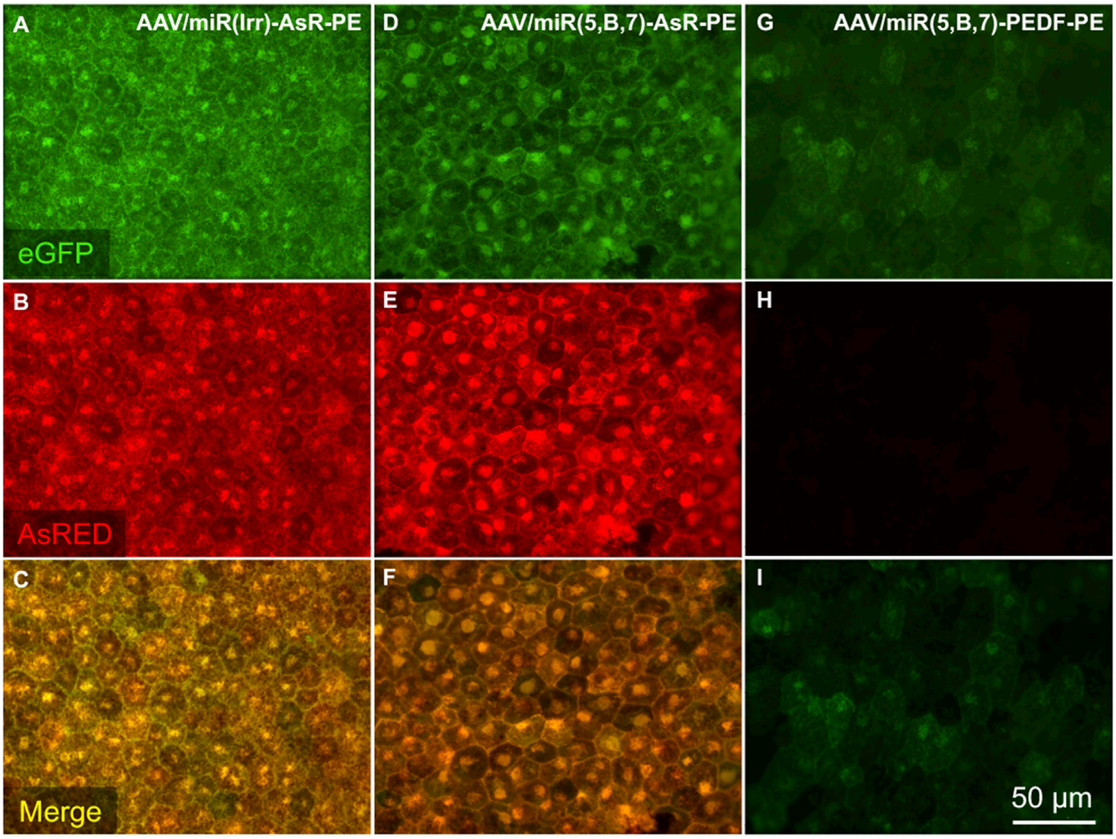

Figure 4. *In Vivo* Expression of EGFP and AsRED following Subretinal Administration of AAV/miR(5,B,7)-AsR-PE, Assessed 57 dpi

Mice were injected with 4.2×10^9 vg AAV/miR(Irr)-AsR-PE (A–C), AAV/miR(5,B,7)-AsR-PE (D–F), and AAV/miR(5,B,7)-PEDF-PE (G–I). 57-dpi mice were euthanized and eyes were enucleated. Following removal of the neuroretina from the eye cup, the RPE cell layer was exposed, and flat-mounts were prepared as described in the Materials and Methods. Representative images of flat-mounts from each group of injections showing whether EGFP (green) and AsRED (red) are expressed *in vivo* were obtained using fluorescence microscopy. Combined images (C, F, and I) were obtained by merging the respective images shown in (A and B), (D and E), and (G and H). Scale bar, 50 μm AsRED (AsR), red fluorescent marker protein; vg, vector genomes.

injection. In the present study, we have refined the multigenic vector even further, by replacing one of the reporter genes with PEDF, and subcloned the entire expression cassette into an AAV plasmid for the subsequent production of clinically applicable AAV vectors. In this study, we show that incorporation of the multigenic expression cassette into the AAV plasmid leads to the following: (1) expression of multiple proteins, including PEDF, *in vitro* following the transfection of relevant mammalian cells; (2) a high level of reporter gene expression in RPE cells following a single subretinal injection in mice; (3) co-expression of miRNA(5,B,7) and PEDF in eyes injected with multigenic AAV/miR(5,B,7)-PEDF-PE vectors; (4) reduced CNV formation in eyes treated with AAV vectors encoding therapeutic cassettes (AAV/miR(5,B,7)-AsR-PE and AAV/miR(5,B,7)-PEDF-PE); and (5) an increased, albeit not statistically significant, efficacy due to combined expression of multiple antiangiogenic molecules (anti-VEGFA miRNAs and PEDF) from the same vector.

The most remarkable finding was a significant reduction of CNV area by 34% and 45% in the eyes of mice treated with AAV/miR(5,B,7)-AsR-PE or AAV/miR(5,B,7)-PEDF-PE, respectively, when compared to control mice injected with AAV/miR(Irr)-AsR-PE particles. Mice were injected subretinally with multigenic AAV particles, and subsequent fluorescence fundoscopy of GFP expression from the vector revealed the transduced area. In a previous study,[36] we argued that local VEGFA levels would be markedly lower in the transduced area due to VMD2-driven anti-VEGFA miRNA expression in RPE cells, and, based on this, we induced CNV via laser burns within the transduced area. This strategy was later deemed suboptimal, since occasionally only small areas were transduced. This made it difficult to distribute the optimal amount of laser burns appropriately, consequently burning off patches of anti-VEGFA miRNA-expressing RPE cells by use of the laser. Furthermore, the close proximity of laser burns to injection-induced retinal damage led to the exclusion of several burns in the study.[36] Hence, in the current study, laser burns were applied outside the transduced area as opposed to the previous study. In the former study, where mice were injected subretinally with LVs harboring the multigenic expression cassette encoding the anti-VEGFA miRNAs (LV/miR(5,B,7)-AsR-PE), the CNV area was

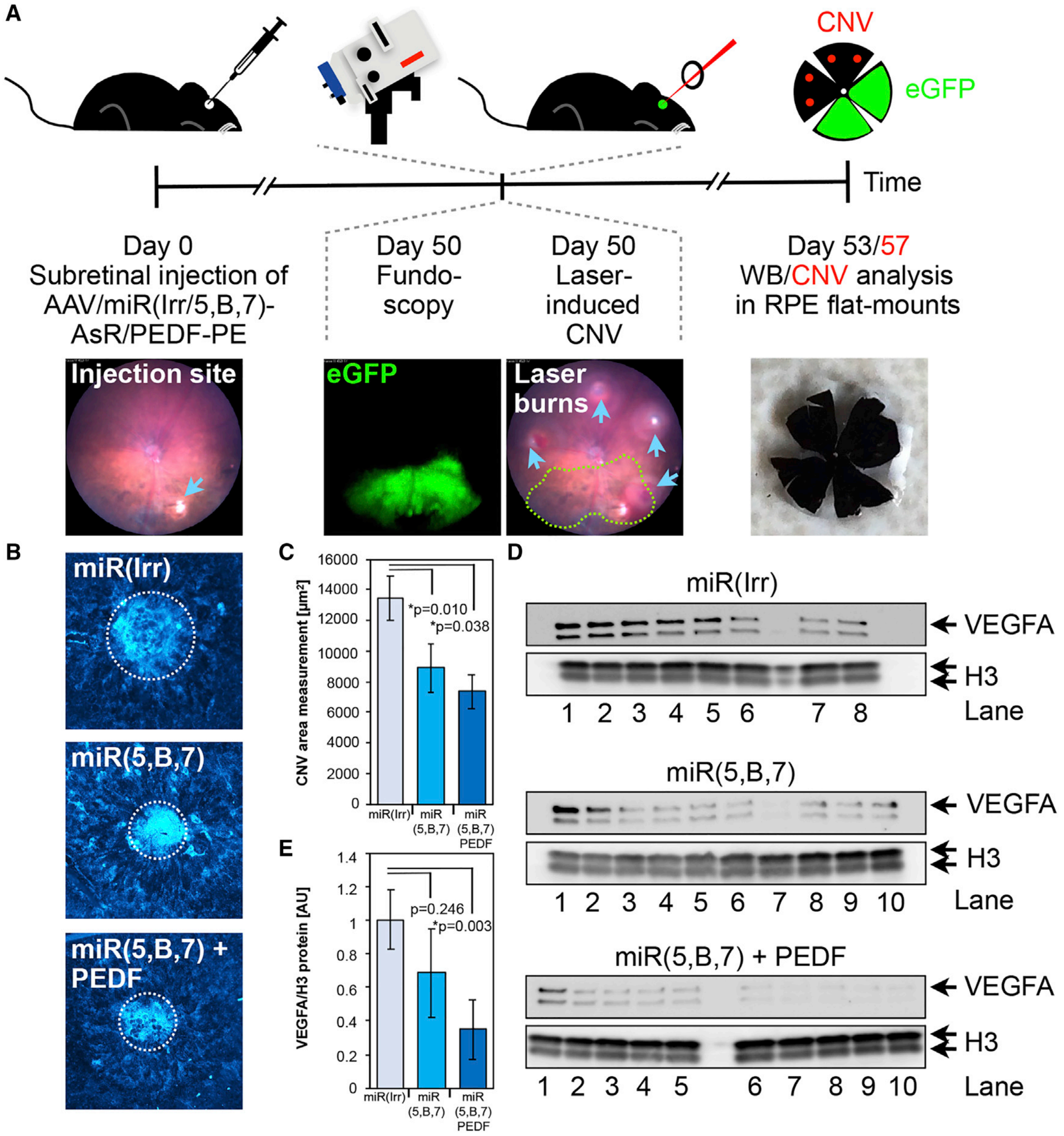

Figure 5. AAV Vectors Expressing the Antiangiogenic Protein PEDF and miRNAs Targeted to *VEGFA* Provide Combined Efficacy

(A) Study design and timeline of the experimental setup used to assess the *in vivo* efficacy of AAV/miR(5,B,7)-PEDF-PE and AAV/miR(5,B,7)-AsR-PE compared to the AAV/miR(Irr)-AsRED-PE. At day 0, the AAV vectors were administrated subretinally to the animals. Three groups of 20 mice received 4.2 × 10^9 vg unilaterally of AAV/miR(Irr)-AsRED-PE, AAV/miR(5,B,7)-AsR-PE, or AAV/miR(5,B,7)-PEDF-PE. At 50 dpi, fluorescence fundoscopy was utilized to assess the EGFP expression (Figures 3 and S2). In addition, mice were treated with an image-guided laser to induce CNV. Four (for CNV area measurements) or 10 (for western blot) laser burns were performed in areas of the retina preferentially in close vicinity to areas with EGFP-positive cells, following previously described recommendations (see the Materials and Methods). At 53 and 57 dpi, retinal tissue was obtained for western blotting (whole eye) and CNV size analysis in flat-mounts, respectively. Illustrative images showing injection site, fundoscopy revealing EGFP expression, CNV burns (blue arrowheads, three burns outside the area showing EGFP-positive cells and one burn at the boundary of the area) in the retina (the area with EGFP expression from the fundoscopy analysis is depicted with green, dotted lines), and a flat-mount. (B) Representative images of CNV following subretinal injection of AAV/miR(Irr)-AsR-PE (miR(Irr)), AAV/miR(5,B,7)-AsR-PE (miR(5,B,7)), and AAV/miR(5,B,7)-PEDF-PE (miR(5,B,7) + PEDF) and subsequent laser-induced CNV and isolectin staining of flat-mounts. The measured CNV areas are highlighted with white dotted lines. (C) CNV area measurements, with "n" representing the number of laser burns assayed, were as following (mean ± SEM): AAV/miR(Irr)-AsR-PE, 1.35 ± 0.14 × 10^4 μm^2 (n = 33); AAV/-miR(5,B,7)-AsR-PE, 0.89 ± 0.16 × 10^4 μm^2 (n = 22); and AAV/-miR(5,B,7)-PEDF-PE, 0.73 ± 0.11 × 10^4 μm^2 (n = 12). Error bars indicate SEM. Statistical differences among the three groups were evaluated after a y = log(y) transformation of the data to obtain Gaussian distributions and then using a one-way ANOVA followed by multiple comparisons (Tukey's multiple comparison test) for miR(5,B,7) versus miR(Irr) p = 0.01 and for miR(5,B,7) + PEDF versus miR(Irr) p = 0.038. *Statistically significant. (D) Western blot analysis of 15 μg total protein extract from whole eyes

(legend continued on next page)

reduced by 85% compared to control mice (injected with LV/miR (Irr)-AsR-PE). Even though the reduction of both CNV area and the total level of VEGFA seemed to be more efficient in the previous study, direct comparison is problematic. Differences in both infectious titer of the two different viral vectors (AAV versus LV) and the applied methodology (laser burns inside versus outside the transduced area) may have affected the results.

Functional expression from the multigenic AAV vectors was further investigated by examining total VEGFA and PEDF levels in the eye. The VEGFA knockdown efficacy of the anti-VEGFA miRNAs and PEDF was assessed by the quantification of total VEGFA levels in laser-treated mouse eyes. Anti-VEGFA miRNAs were able to reduce VEGFA levels by 32%, whereas anti-VEGFA miRNAs combined with the expression of PEDF significantly reduced VEGFA levels by 65%. The PEDF expression and the level of *VEGFA* mRNA were analyzed in animals subjected only to subretinal injection of AAV vectors. As expected, robust expressions of *PEDF* mRNA and PEDF protein were observed in animals receiving AAV/miR(5,B,7)-PEDF-PE. Furthermore, a significant reduction of *VEGFA* mRNA expression, as a consequence of PEDF and miRNA(5,B,7) co-expression, was found in this group. Collectively, these results demonstrate that both factors of the dual-acting AAV/miR(5,B,7)-PEDF-PE vector are expressed in RPE cells in the mouse retina and that combining anti-VEGFA miRNAs and PEDF expression in RPE cells in mice has a joint effect on the reduction in total VEGFA in the eye. PEDF is an endogenously expressed antiangiogenic protein, which is secreted by cells of the retina and choroid. PEDF does not directly interact with VEGFA, but it has been found to inhibit VEGFA expression at the transcriptional level, since PEDF inhibits hypoxia-induced increases in *VEGFA* promoter activity, HIF-1 nuclear translocation, and mitogen-activated protein kinase phosphorylation.[52] Even though the assessment of VEGFA relies on 10 laser burns (compared to 4 burns in the case of CNV size evaluation),[36,47–49] the presented results clearly show that the VEGFA level is reduced in eyes treated with either anti-VEGFA miRNAs or anti-VEGFA miRNAs and PEDF.

Whereas most angiogenic factors stimulate the early stages of angiogenesis, Angpt-1 modulates late stages, including vascular maturation and remodeling.[53] To demonstrate the antiangiogenic effect of anti-VEGF therapy in general, we therefore investigated the expression of *Angpt-1* in the retina following subretinal delivery of AAV/miR(5,B,7)-PEDF-PE. The suppression of *VEGFA* resulted in a significant reduction of *Angpt-1* compared to control eyes. This is in line with previous findings showing increased *Angpt-1* mRNA in a time- and dose-dependent manner upon VEGF stimulation in human RPE cells.[50] Similarly, Zadeh and colleagues[54] found *Angpt-1* expression to be significantly diminished in a tumor angiogenesis model, following the downregulation of *VEGFA*. Even though we cannot formally rule out that injection of the control AAV vector (AAV/miR(Irr)-AsR-PE) might have some impact, the data strongly suggest that our anti-VEGF therapy affects other players in the angiogenic pathway, following reduced *VEGFA* expression.

The multigenic AAV vectors were packaged in serotype 5 capsids based on its RPE tropism and a study done by the Auricchio group[55] showing a serotype-dependent packaging of large genes in AAV vectors. This study showed that serotype 5 was able to tolerate the large murine *ABCA4* and human *MYO7A* and *CEP290* genes better when compared to other AAV serotypes with RPE tropism, such as serotype 1, 2, 4, 7, 8, and 9. Furthermore, serotype 5 improved the ocular phenotype in $ABCA4^{-/-}$ mice following an intraocular administration of AAV particles encapsulating oversized *ABCA4*-encoding genomes (8.9 kb). Our findings unveil a slight variation in transduction efficiencies of the three different AAV vectors, as illustrated by Figure 4, where equivalent amounts of vector genomes were injected into the mice. Although we cannot at present explain such variation, it seems plausible that titers are affected by the size of the construct being packaged. Mice injected with AAV/miR(5,B,7)-PEDF-PE (5.3 kb) had a markedly lower expression of EGFP in the RPE cells of the isolated RPE and choroidal flat-mounts when compared to RPE and choroidal flat-mounts from mice injected with AAV/miR(Irr)-AsR-PE and AAV/miR(5,B,7)-AsR-PE (both 4.7 kb). Hence, the observed difference in EGFP expression (compare Figure 4G with Figures 4A and 4D) does not reflect reduced cell density in RPE and choroidal flat-mounts from mice injected with AAV/miR(5,B,7)-PEDF-PE, but rather a reduced number of transduced cells.

This suggests that oversized genomes are truncated, which has previously been proposed by other groups.[17,55–57] Wu et al.[17] reported that packaged vector genomes in serotype 5 never exceeded 5.2 kb and that longer packaged vector genomes existed as a mixture of heterogeneously sized genomes, i.e., a mixture of fragmented AAV (fAAV) vectors. Vector preparations containing fAAV vectors feature genomes of varying length and polarity, and transduction of single cells with two fAAV vectors may lead to hybridization of overlapping segments and subsequent host-cell DNA repair to form a full-length transgene.[58,59] These findings may suggest that our AAV/miR(5,B,7)-PEDF-PE vector preparation is a fAAV mixture, whereas AAV/miR(-Irr)-AsR-PE and AAV/miR(5,B,7)-AsR-PE vectors are packaged as full-size genomes. Also, this could potentially explain the difference in EGFP expression levels in RPE cells, despite the fact that equimolar amounts of vector genomes were injected per mouse. Furthermore, optimization of the multigenic expression cassette to fit properly

transduced with 4.2×10^9 vg unilaterally of AAV/miR(Irr)-AsR-PE (top panel lanes 1–8), AAV/miR(5,B,7)-AsR-PE (middle panel lanes 1–10), or AAV/-miR(5,B,7)-PEDF-PE (bottom panel lanes 1–10). CNV was induced by laser 50 dpi, and eyes were isolated 3 days later. The cell lysates were subjected to electrophoresis, blotting, and immunostaining using a rabbit anti-VEGF antibody. As a loading control for the cell lysate samples, a rabbit antibody against H3 was used. The positions of VEGFA and H3 are indicated with arrows. (E) Assessment of the VEGFA levels in extracts from whole eyes analyzed by western blotting as shown in (D). Error bars indicate SEM. Statistical differences among the three groups were evaluated after a y = log(y) transformation of the data to obtain Gaussian distributions and then using a one-way ANOVA, for miR(5,B,7) + PEDF versus miR(Irr) p = 0.003. *Statistically significant. CNV, choroidal neovascularization; H3, histone H3; vg, vector genomes.

into AAV capsids would most likely improve the effect of combining anti-VEGFA miRNAs and PEDF. A possible way to optimize the multigenic expression cassette is to replace the woodchuck hepatitis virus posttranscriptional regulatory element (WPRE) in the GFP expression cassette with a poly(A) signal, thereby trimming 0.4 kb off the construct. *In vitro* transfection of human cell lines with such constructs indicates efficient and even significantly higher GFP expression in HEK293 cells and concomitant PEDF expression in melanoma cells (Figure S4). Additional trimming of base pairs could also be done by optimizing the VMD2 promoter sequence.

Collectively, the dual-acting AAV/miR(5,B,7)-PEDF-PE vector showed therapeutic efficacy based on joint expression of two antiangiogenic effectors. Although differences in therapeutic efficacy between mice treated with AAV/miR(5,B,7)-PEDF-PE and the group receiving AAV/miR(5,B,7)-AsR-PE did not reach statistical significance, the dual-acting vector showed efficacy despite being affected in functional titers leading to lower expression of EGFP in the RPE cells, as described above. This strongly suggests that co-delivery of PEDF improves therapeutic efficacy substantially. In the present setup, it is not possible to determine if the effect on reducing VEGFA expression in mice treated with AAV/miR(5,B,7)-PEDF-PE is caused prominently by miR(5,B,7) or by PEDF. However, as PEDF is a secretory, antiangiogenic protein involved in neuroprotection, angiogenesis, and inflammation, we may speculate that this factor has the strongest therapeutic impact overall in the retina.[38–42] On the other hand, miR(5,B,7) may have the strongest local effect on reducing VEGFA expression, thereby contributing to the measured antiangiogenic effect, as this factor targets *VEGFA* mRNA expressed in transduced RPE cells.

To ensure delivery of the antiangiogenic agents with an RPE cell-specific expression pattern, the expression cassette was driven by the VMD2 promoter. As expected, robust expression within the RPE cells was observed. This is in line with our previous findings showing RPE-restricted expression from the delivered VMD2-driven cassette.[34] Using a hypoxia response element (HRE), a recent paper demonstrated effective HRE-driven expression in RPE cells during hypoxic conditions.[60] Introduction of such a regulatory element into the multigenic vector may further enhance the efficacy and fine-tune the expression of antiangiogenic molecules, especially for prophylactic application.

The present study represents the first attempt to test a multigenic AAV system. An important benefit of our multigenic AAV vector is the potential for simultaneous expression of multiple antiangiogenic factors, enabling cell-specific targeting of different pathways involved in the pathogenesis of AMD. Currently available therapies focus on reducing the VEGFA levels, without addressing other aspects of the AMD pathophysiology. By demonstrating that dual-acting gene therapy targeting VEGFA delivered by multigenic AAV vectors evidently provides combined efficacy, we believe that multigenic AAV vectors may be an important tool in the future treatment of neovascular diseases of the retina, including AMD. Hence, such vectors can be utilized to deliver either a therapeutic cocktail of antiangiogenic molecules or a combination of angiostatic proteins and molecules designed to dampen other nAMD-related pathological changes, including inflammation within the retina.

MATERIALS AND METHODS

Vector Construction

To generate p/miR(5,B,7)-AsR-PE, the multigenic cassette, which was originally designed for expression in an LV vector,[34,35] was inserted into the pAAV/siRNA (Applied Viromics, Freemont, CA, USA), which was cleaved with the *Asc*I and *Spe*I restriction enzymes. The sequence containing the multigenic cassette was amplified in two separate PCR reactions with Phusion Hot Start Flex 2X Master Mix (New England Biolabs, Bionordika, Herlev, Denmark), with primers containing an overlap region of 20 bp complementary to the adjacent fragment in the final construct as well as relevant restriction sites. PCR products were purified using MinElute PCR purification kit (QIAGEN, Copenhagen, Denmark) or on a 1% agarose gel followed by extraction using the Qiaquick gel extraction kit (QIAGEN). The two fragments and the cleaved vector were assembled using the NEBuilder Master Mix (New England Biolabs), according to the manufacturer's protocol. The fragments were incubated for 60 min at 50°C. 0.125 pmol DNA was used in a 20-μL reaction with a 1:2 vector-to-insert ratio for each insert. As an irrelevant, nontargeting negative control, p/miR(Irr)-AsR-PE was generated by subcloning of the miRNA cluster containing the miR(Irr) from the multigenic LV expression cassette[34,35] into the p/miR(5,B,7)-AsR-PE, after removal of miR(5,B,7) with *Bsi*WI and *Nsi*I. The miR(Irr) contains the HIV-targeting MCM7-based miRNA cluster, miR(S1,S2,S3).[61] p/miR(5,B,7)-PEDF-PE was generated by cleaving the fragment containing human PEDF and part of the intron sequence containing miR(5,B,7) from the multigenic LV expression cassette[34] with *Xba*I and inserted into p/miR(5,B,7)-AsR-PE using standard cloning methods.

p/miR(Irr)-AsR-PE-PolyA, p/miR(5,B,7)-AsR-PE-PolyA, and p/miR(5,B,7)-PEDF-PE-PolyA were generated by amplification of the PGK-EGFP-BGH poly(A) fragment from the pFRT/EGFP-PGK plasmid in a PCR reaction with Phusion Hot Start polymerase (New England Biolabs), introducing a *Cla*I restriction at the poly(A) end, and purified using the minElute PCR purification kit (QIAGEN). The fragment was inserted into *Kpn2*I- and *Cla*I-cleaved p/miR(Irr)-AsR-PE, p/miR(5,B,7)-AsR-PE, and p/miR(5,B,7)-PEDF-PE vectors.

For all cloning procedures, vector fragments were treated with 2 μL phosphatase (FastAP, Thermo Fisher Scientific, Hvidovre, Denmark) in a reaction volume of 100 μL for 30 min at 37°C after restriction digest, and the vector was purified on a 1% agarose gel and extracted using the Qiaquick gel extraction kit (QIAGEN).

Cell Culture, Transfection, and Transduction

HEK293 (CRL-1573; American Type Culture Collection, Manassas, VA) and human melanoma cells[62] were kept as previously described[34] in T-75 cell culture flasks (Sarstedt, Nümbrecht, Germany). For melanoma cells, transfections were performed with Turbofect (Thermo Fisher Scientific), using 0.547 pmol DNA for each well in a 6-well plate or for each slide flask and a DNA-to-transfection

reagent ratio of 1 μg:2 μL, according to the manufacturer's protocol. Medium was replaced 24 h post-transfection. HEK293 cells were transfected using X-tremeGENE HP DNA Transfection Reagent (Roche Diagnostics, Hvidovre, Denmark), according to the manufacturer's protocol, using 0.547 pmol DNA per well in 6-well plates and a DNA-to-transfection reagent ratio of 1 μg:2 μL.

Flow Cytometry Analysis

HEK293 cells were analyzed for GFP expression approximately 48 h post-transfection. Before flow cytometry analysis, cells were trypsinized, washed, and resuspended in PBS. Flow cytometry analysis was performed using a NovoCyte Flow Cytometer (Acea Biosciences, San Diego, CA, USA) and analyzed with FlowJo (Tree Star, Ashland, OR, USA).

Immunostaining of PEDF *In Vitro*

Melanoma cells for immunostaining were kept in SlideFlasks (Thermo Fisher Scientific). Cells were fixed and immunostained as described previously for HEK293 cells.[34] Mouse anti-PEDF-antibody (MAB1059; Millipore, Hellerup, Denmark) was diluted 1:100, while the secondary antibody (568 Alexa Fluor Goat-anti-mouse, Thermo Fisher Scientific) was diluted 1:400 both in PBS with 0.5 w/v% BSA. Nuclei were stained with DAPI (Sigma-Aldrich).

Microscopy

Immunostained PEDF in melanoma cells was visualized using a confocal laser-scanning microscope (CLSM) (LSM 710; Zeiss, Jena, Germany), as previously described.[34] Images of melanoma cells as well as RPE cells in flat-mounts (*in vivo*) expressing EGFP and AsRED were captured by a fluorescence microscope (Leica DM IRBE), equipped with a Leica DFC 360 FX camera (both Leica Microsystems, Wetzlar, Germany).[36]

AAV Production

AAV vectors encapsulated in serotype 5 capsids were produced at The University of North Carolina Gene Therapy Center (UNC) Vector Core (Chapel Hill, NC, USA), using the three different vector plasmids: pAAV/miR(Irr)-AsR-PE, pAAV/miR(5,B,7)-AsR-PE, and pAAV/miR(5,B,7)-PEDF-PE. The following titers were obtained: 2.7×10^{12} vg/mL for AAV/miR(Irr)-AsR-PE, 2.1×10^{12} vg/mL for AAV/miR(5,B,7)-AsR-PE, and 2.3×10^{12} vg/mL for AAV/miR(5,B,7)-PEDF-PE.

Animals

8-week-old C57BL/6J male mice were purchased from Janvier Labs (Le Genest-Saint-Isle, France). The mice were housed at the animal facilities (Department of Biomedicine, Aarhus University, Denmark) and kept on a 12-h light/12-h dark cycle. Mice were anesthetized with a combination of ketamine and medetomidin hydrochloride (Ketador 60–100 mg/kg; Richter Pharma, Wels, Austria, and Cepetor 0.5–1 mg/kg; ScanVet Animal Health, Fredensborg, Denmark) prior to subretinal injection, laser treatment to induce CNV, or *in vivo* fundus photography. Pupils were dilated with a drop of 1% tropicamide solution (Mydriacyl; Alcon Nordic, Copenhagen, Denmark) and Metaoxedrin 10% (Skanderborg Apotek, Skanderborg, Denmark). During anesthesia, eyes were lubricated with a carbomer eye gel (Viscotears 2 mg/g; Alcon Nordic). Immediately after the investigations, mice were brought out of anesthesia with Antisedan 0.5–1 mg/kg, and they were placed on a warming plate until they moved spontaneously, after which they were transferred to their cages. Mice received a subcutaneous injection of the NSAID carprofen (Norodyl; ScanVet Animal Health) (5 mg/kg) 24 h prior to and immediately after subretinal injection. Additionally, mice were treated with carprofen 5 mg/150 mL via their drinking water 1 day prior to and 3 days after AAV administration. All animal experiments were performed under the approval of The Danish Animal Inspectorate (case 2015-15-0201-00691).

Subretinal Injections

Mice were injected with approximately 4.2×10^9 vg in a total volume of 2 μL, in accordance with the method described by Bemelmans et al.[63] Three groups of 20 mice were injected unilaterally with AAV/miR(Irr)-AsR-PE, AAV/miR(5,B,7)-AsR-PE, or AAV/miR(5,B,7)-PEDF-PE. For the analysis of the expression of *VEGFA*, *PEDF*, and *Angpt-1 in vivo*, 16 additional mice were unilaterally injected with 4.2×10^9 vg AAV/miR(5,B,7)-PEDF-PE. In total, five of the 76 treated mice were discarded either due to severe complications following injection (4 animals) or a total lack of EGFP expression examined by fluorescence fundoscopy (1 animal).

Laser-Induced CNV

Laser induction of CNV in mice was performed 50 days after vector injection via an image-guided laser system, the Micron IV (Phoenix Research Laboratories, Pleasanton, CA), in accordance with the method described by Gong et al.[64] The laser settings were as follows: wavelength, 532 nm, 240 mW; duration, 70 ms; and size, 50 μm. In mice intended for subsequent CNV area measurements on RPE and choroidal flat-mounts, the laser was applied 4 times to each injected eye in the untransduced area or in the periphery of the transduced area, as visualized by fluorescence fundus imaging (Micron IV, Phoenix Research Laboratories). The distance between two laser burns as well as between a laser burn and the optic nerve was approximately double the disc diameter of the optic nerve. Mice intended for western blot analysis of intraocular VEGFA levels received 10 laser burns in the untransduced area or in the periphery of the transduced area, as visualized by fluorescence fundus imaging (Micron IV, Phoenix Research Laboratories), as previously described.[36] No significant change in VEGFA levels in the eye could be detected by western blotting following 4 laser burns (data not shown).

Immunostaining of RPE and Choroidal Flat-Mounts

AAV-treated C57BL/6J mice were sacrificed 7 days after laser-induced CNV. Their eyes were enucleated, placed in a freshly made 4% paraformaldehyde (PFA) solution, and fixated for 2 h at room temperature. Dissection was carried out as described by Askou et al.[36] Flat-mounts were transferred to a 96-well plate where immunostaining with isolectin, *Griffonia simplicifolia* (GS)-IB_4 (biotin conjugated, I21414; Thermo Fisher Scientific), was carried out as described by Askou et al.,[36] and, finally, flat-mounts were gently transferred to Super-FrostPlus glass slides (Menzel-Glaser,

Braunschweig, Germany) and mounted using ProLong Gold antifade reagent (Life Technologies, Taastrup, Denmark).

CNV Area Measurements

The size of the CNV was measured on GS-IB_4-stained flat-mounts. Eyes with laser-induced CNV were analyzed for EGFP and AsRED expression, as well as for CNV by fluorescence microscopy (Leitz DM RB; Leica Microsystems). Images were captured with a Leica DFC 360 FX camera and associated software (Leica Application Suite version 3, Leica Microsystems). Computer-assisted image analysis software (ImageJ; https://imagej.nih.gov/ij/) was used to measure the CNV area in the following manner: images of laser burns were loaded into the software, and, after a thorough evaluation of the outline of each laser burn, it was deemed appropriate to use the "oval" tool to encompass the CNV. The number of pixels within the area covered by the oval was then measured. This process was performed on all laser burns deemed appropriate for analysis, following the recommendations of Gong et al.[64]

Quantification of EGFP Expression *In Vivo*

EGFP expression, visualized by fluorescence fundus imaging using the Micron IV (Phoenix Research Laboratories), was quantified using ImageJ.

Western Blotting

For the analysis of PEDF in transfected cells, cells and media were harvested for western blotting 72 h post-transfection, as described previously;[65] however, the cells were lysed in a volume of 150 μL, lysates were sonicated for 1 min at a Bioruptor (Diagenode, Liège, Belgium) and centrifuged at 14,000 × *g* for 15 min, and the media were filtered (0.20 μm). Total protein concentration was measured using the Protein Assay Dye Reaction Concentrate (Bio-Rad, Hercules, CA, USA), according to the manufacturer's protocol. 40 μg total protein and 36 μL media were loaded onto the gel, and western blotting was performed as previously described.[36]

For VEGFA analysis in whole eyes, mice were sacrificed 3 days after laser-induced CNV. The eyes were extracted and placed immediately in ice-cold PBS (Sigma-Aldrich), and excessive periocular tissue was removed. The eye was processed according to the MicroRotofor (Bio-Rad) protocol but with the use of radioimmunoprecipitation assay (RIPA) buffer (Thermo Fisher Scientific) with added proteinase inhibitor (complete Mini, Roche Diagnostics) instead of the protein solubilization buffer supplied within the kit.

For analysis of *in vivo* expression of PEDF in the retina, mice were sacrificed 28 dpi of 4.2 × 10^9 vg AAV/miR(5,B,7)-PEDF-PE. The eyes were extracted, placed immediately in ice-cold Hank's balanced salt solution (HBSS, Sigma-Aldrich), and dissected under a microscope, as described previously.[36] In short, excessive periocular tissue was removed, followed by removal of the cornea and lens using Vannas scissors and, lastly, gentle removal of the neuroretina with forceps. The neuroretina was placed in 50 μL RIPA buffer on dry ice for 3 min followed by 3 min at 37°C and vortexing. This was repeated three times. Subsequently, the neuroretina was incubated at 4°C with shaking overnight in RIPA buffer. The following day, cell debris was sedimented by centrifugation at full-speed for 15 min at 4°C, and the supernatant was recovered.

Total protein concentration was measured using the Protein Assay Dye Reaction Concentrate (Bio-Rad), according to the manufacturer's protocol. Western blotting was performed on the lysate (for PEDF analysis) or the grinded lysate (for VEGFA analysis), as described previously.[36] Membranes were incubated at 4°C overnight with rabbit anti-VEGF antibody (ab46154; Abcam, Cambridge, UK) or monoclonal mouse anti-PEDF antibody (MAB1059, Millipore) in a concentration of 1:1,000. Washing and blocking of anti-PEDF was done in accordance with the protocol from Millipore. As a loading control, membranes were incubated for 1 h at room temperature with either polyclonal rabbit anti-histone H3 (ab1791, Abcam) or monoclonal mouse anti-vinculin antibody (V91131, Sigma-Aldrich) in a concentration of 1:10,000. Membranes were then washed three times in Tris-buffered saline, 0.1% Tween 20 (TBS-T), and subsequently they were incubated with horseradish peroxidase (HRP)-conjugated goat-anti-rabbit or goat-anti-mouse antibodies (Dako; Agilent Technologies, Santa Clara, CA, USA) for 1 h at room temperature (RT). Bound antibodies were visualized with Clarity Western ECL Blotting substrate (Bio-Rad) on an ImageQuant LAS4000 digital imaging system (GE Healthcare, Cleveland, OH), and densitometric quantification of the bands was performed using ImageJ or Image Lab version 6.0.1 (Bio-Rad).

RNA Extraction, cDNA Synthesis, and Real-Time qPCR Analysis of VEGFA, PEDF, and Angpt-1 and RT-PCR Analysis of the pri-miR(5,B,7) Transcript Expression *In Vivo*

28 dpi of 4.2 × 10^9 vg AAV/VMD2-miR(5,B,7)-PEDF-PE, mice were sacrificed by cervical dislocation and eyes were enucleated and dropped in ice-cold HBSS buffer (Sigma-Aldrich). Periocular tissue was removed and the anterior segment and lens were discarded. The neuroretina was removed and the remaining eye cup was placed in 200 μL ice-cold RNAprotect cell Reagent (QIAGEN). The tube was vortexed every 2 min for 10 min to aid the release of RPE cells. The eye cup was removed and cells were pelleted at 5,000 × *g* for 5 min and the RNAprotect cell reagent was removed. The pellet was resuspended in RLT buffer and RNA was purified using the RNeasy plus micro kit, according to protocol (QIAGEN). The iScript cDNA synthesis kit (Bio-Rad) was used for first-strand cDNA synthesis, with a total input RNA of 50 ng, according to the protocol. PCR reactions were performed in triplicate on 1:5 diluted cDNA using LightCyclerW480 SYBR Green I Master (Roche Diagnostics) and analyzed on a LightCyclerW480 (Roche Diagnostics). Relative gene expression of *VEGFA*, *PEDF*, and *Angpt-1* was calculated using the standard curve method[66] and related relative to the endogenous control, the housekeeping gene hypoxanthine-guanine phosphoribosyl transferase (*HPRT*) mRNA. To investigate expression of the pri-miR(5,B,7) transcript, cDNA was amplified by standard PCR and analyzed by agarose gel-electrophoresis. The contralateral, uninjected eye was used as a control.

All primer sequences and PCR conditions are available upon request.

Statistical Analysis

Data are presented as the mean ± SEM, unless otherwise stated. Statistical differences between two groups were evaluated using Student's t test, unless otherwise stated. Statistical differences among three groups were evaluated using one-way ANOVA for the comparison of multiple groups, unless otherwise stated. A p value of < 0.05 was considered statistically significant.

SUPPLEMENTAL INFORMATION

Supplemental Information includes four figures and can be found with this article online at https://doi.org/10.1016/j.omtn.2019.01.012.

AUTHOR CONTRIBUTIONS

Writing – Original Draft, A.L.A. and T.J.C.; Writing – Reviewing and Editing, A.L.A., S.A., T.B., L.A., J.G.M., and T.J.C.; Supervision, T.J.C. and A.L.A.; Investigation, A.L.A., S.A., J.N.E.B., and A.H.; Methodology, T.J.C., A.L.A., T.B., L.A., and J.G.M.

CONFLICTS OF INTEREST

The authors have no conflicts of interest.

ACKNOWLEDGMENTS

The authors would like to thank Tina Hindkjaer and Kamilla Zahll Hornbek for their excellent technical support. This work was supported by the Faculty of Health Sciences (PhD scholarship to S.A. and A.H.), the Danish Council for Independent Research (T.J.C., grant 4183-00017B), the Gene Therapy Initiative Aarhus (GTI-Aarhus) funded by the Lundbeck Foundation (T.J.C., L.A., and J.G.M., grant R126-2012-12456), the Lundbeck Foundation (A.L.A., grant R165-2013-15631), the Danish Eye Research Foundation (T.J.C.), Aase og Ejnar Danielsen's Foundation (T.J.C.), Købmand Marie Kirstine Jensens Fond (T.B.), the Riisfort Foundation (T.J.C.), and Svend Helge Schrøder og hustru Ketty Lydia Larsen Schrøders fond (T.J.C.).

REFERENCES

1. Bainbridge, J.W., Smith, A.J., Barker, S.S., Robbie, S., Henderson, R., Balaggan, K., Viswanathan, A., Holder, G.E., Stockman, A., Tyler, N., et al. (2008). Effect of gene therapy on visual function in Leber's congenital amaurosis. N. Engl. J. Med. *358*, 2231–2239.
2. Hauswirth, W.W., Aleman, T.S., Kaushal, S., Cideciyan, A.V., Schwartz, S.B., Wang, L., Conlon, T.J., Boye, S.L., Flotte, T.R., Byrne, B.J., and Jacobson, S.G. (2008). Treatment of leber congenital amaurosis due to RPE65 mutations by ocular subretinal injection of adeno-associated virus gene vector: short-term results of a phase I trial. Hum. Gene Ther. *19*, 979–990.
3. Maguire, A.M., Simonelli, F., Pierce, E.A., Pugh, E.N., Jr., Mingozzi, F., Bennicelli, J., Banfi, S., Marshall, K.A., Testa, F., Surace, E.M., et al. (2008). Safety and efficacy of gene transfer for Leber's congenital amaurosis. N. Engl. J. Med. *358*, 2240–2248.
4. Nathwani, A.C., Tuddenham, E.G., Rangarajan, S., Rosales, C., McIntosh, J., Linch, D.C., Chowdary, P., Riddell, A., Pie, A.J., Harrington, C., et al. (2011). Adenovirus-associated virus vector-mediated gene transfer in hemophilia B. N. Engl. J. Med. *365*, 2357–2365.
5. Corti, M., Liberati, C., Smith, B.K., Lawson, L.A., Tuna, I.S., Conlon, T.J., Coleman, K.E., Islam, S., Herzog, R.W., Fuller, D.D., et al. (2017). Safety of Intradiaphragmatic Delivery of Adeno-Associated Virus-Mediated Alpha-Glucosidase (rAAV1-CMV-hGAA) Gene Therapy in Children Affected by Pompe Disease. Hum. Gene Ther. Clin. Dev. *28*, 208–218.
6. Rafii, M.S., Baumann, T.L., Bakay, R.A., Ostrove, J.M., Siffert, J., Fleisher, A.S., Herzog, C.D., Barba, D., Pay, M., Salmon, D.P., et al. (2014). A phase1 study of stereotactic gene delivery of AAV2-NGF for Alzheimer's disease. Alzheimers Dement. *10*, 571–581.
7. Ghazi, N.G., Abboud, E.B., Nowilaty, S.R., Alkuraya, H., Alhommadi, A., Cai, H., Hou, R., Deng, W.T., Boye, S.L., Almaghamsi, A., et al. (2016). Treatment of retinitis pigmentosa due to MERTK mutations by ocular subretinal injection of adeno-associated virus gene vector: results of a phase I trial. Hum. Genet. *135*, 327–343.
8. LaVail, M.M., Yasumura, D., Matthes, M.T., Yang, H., Hauswirth, W.W., Deng, W.T., and Vollrath, D. (2016). Gene Therapy for MERTK-Associated Retinal Degenerations. Adv. Exp. Med. Biol. *854*, 487–493.
9. MacLaren, R.E., Groppe, M., Barnard, A.R., Cottriall, C.L., Tolmachova, T., Seymour, L., Clark, K.R., During, M.J., Cremers, F.P., Black, G.C., et al. (2014). Retinal gene therapy in patients with choroideremia: initial findings from a phase 1/2 clinical trial. Lancet *383*, 1129–1137.
10. Patrício, M.I., Barnard, A.R., Xue, K., and MacLaren, R.E. (2018). Choroideremia: molecular mechanisms and development of AAV gene therapy. Expert Opin. Biol. Ther. *18*, 807–820.
11. Colella, P., Ronzitti, G., and Mingozzi, F. (2017). Emerging Issues in AAV-Mediated *In Vivo* Gene Therapy. Mol. Ther. Methods Clin. Dev. *8*, 87–104.
12. Daya, S., and Berns, K.I. (2008). Gene therapy using adeno-associated virus vectors. Clin. Microbiol. Rev. *21*, 583–593.
13. Naso, M.F., Tomkowicz, B., Perry, W.L., 3rd, and Strohl, W.R. (2017). Adeno-Associated Virus (AAV) as a Vector for Gene Therapy. BioDrugs *31*, 317–334.
14. Corydon, T.J. (2015). Antiangiogenic Eye Gene Therapy. Hum. Gene Ther. *26*, 525–537.
15. U.S. Food and Drug Administration (2017). FDA approves novel gene therapy to treat patients with a rare form of inherited vision loss. https://www.fda.gov/NewsEvents/Newsroom/PressAnnouncements/ucm589467.htm.
16. Srivastava, A., Lusby, E.W., and Berns, K.I. (1983). Nucleotide sequence and organization of the adeno-associated virus 2 genome. J. Virol. *45*, 555–564.
17. Wu, Z., Yang, H., and Colosi, P. (2010). Effect of genome size on AAV vector packaging. Mol. Ther. *18*, 80–86.
18. Balaggan, K.S., and Ali, R.R. (2012). Ocular gene delivery using lentiviral vectors. Gene Ther. *19*, 145–153.
19. Persons, D.A. (2010). Lentiviral vector gene therapy: effective and safe? Mol. Ther. *18*, 861–862.
20. Yan, Z., Zhang, Y., Duan, D., and Engelhardt, J.F. (2000). Trans-splicing vectors expand the utility of adeno-associated virus for gene therapy. Proc. Natl. Acad. Sci. USA *97*, 6716–6721.
21. McClements, M.E., Barnard, A.R., Singh, M.S., Charbel Issa, P., Jiang, Z., Radu, R.A., and MacLaren, R.E. (2018). An AAV dual vector strategy ameliorates the Stargardt phenotype in adult $Abca4^{-/-}$ mice. Hum. Gene Ther. Published online December 26, 2018. https://doi.org/10.1089/hum.2018.156.
22. Maddalena, A., Tornabene, P., Tiberi, P., Minopoli, R., Manfredi, A., Mutarelli, M., Rossi, S., Simonelli, F., Naggert, J.K., Cacchiarelli, D., and Auricchio, A. (2018). Triple Vectors Expand AAV Transfer Capacity in the Retina. Mol. Ther. *26*, 524–541.
23. Reich, S.J., Auricchio, A., Hildinger, M., Glover, E., Maguire, A.M., Wilson, J.M., and Bennett, J. (2003). Efficient trans-splicing in the retina expands the utility of adeno-associated virus as a vector for gene therapy. Hum. Gene Ther. *14*, 37–44.
24. Gemenetzi, M., and Patel, P.J. (2017). A Systematic Review of the Treat and Extend Treatment Regimen with Anti-VEGF Agents for Neovascular Age-Related Macular Degeneration. Ophthalmol. Ther. *6*, 79–92.
25. Wykoff, C.C., Clark, W.L., Nielsen, J.S., Brill, J.V., Greene, L.S., and Heggen, C.L. (2018). Optimizing Anti-VEGF Treatment Outcomes for Patients with Neovascular Age-Related Macular Degeneration. J. Manag. Care Spec. Pharm. *24* (2-a Suppl), S3–S15.
26. Jaki Mekjavic, P., and Zaletel Benda, P. (2018). Outcome of 5-Year Treatment of Neovascular Age-Related Macular Degeneration With Intravitreal Anti-VEGF Using "Treat and Extend" Regimen. Front. Med. (Lausanne) *5*, 125.
27. Askou, A.L. (2014). Development of gene therapy for treatment of age-related macular degeneration. Acta Ophthalmol. *92 Thesis3*, 1–38.
28. Nowak, J.Z. (2006). Age-related macular degeneration (AMD): pathogenesis and therapy. Pharmacol. Rep. *58*, 353–363.

29. Rofagha, S., Bhisitkul, R.B., Boyer, D.S., Sadda, S.R., and Zhang, K.; SEVEN-UP Study Group (2013). Seven-year outcomes in ranibizumab-treated patients in ANCHOR, MARINA, and HORIZON: a multicenter cohort study (SEVEN-UP). Ophthalmology *120*, 2292–2299.

30. Bhisitkul, R.B., Desai, S.J., Boyer, D.S., Sadda, S.R., and Zhang, K. (2016). Fellow Eye Comparisons for 7-Year Outcomes in Ranibizumab-Treated AMD Subjects from ANCHOR, MARINA, and HORIZON (SEVEN-UP Study). Ophthalmology *123*, 1269–1277.

31. Kauppinen, A., Paterno, J.J., Blasiak, J., Salminen, A., and Kaarniranta, K. (2016). Inflammation and its role in age-related macular degeneration. Cell. Mol. Life Sci. *73*, 1765–1786.

32. Szatmári-Tóth, M., Kristóf, E., Veréb, Z., Akhtar, S., Facskó, A., Fésüs, L., Kauppinen, A., Kaarniranta, K., and Petrovski, G. (2016). Clearance of autophagy-associated dying retinal pigment epithelial cells - a possible source for inflammation in age-related macular degeneration. Cell Death Dis. *7*, e2367.

33. Nguyen, Q.D., Schachar, R.A., Nduaka, C.I., Sperling, M., Klamerus, K.J., Chi-Burris, K., Yan, E., Paggiarino, D.A., Rosenblatt, I., Aitchison, R., and Erlich, S.S.; MONET Clinical Study Group (2012). Evaluation of the siRNA PF-04523655 versus ranibizumab for the treatment of neovascular age-related macular degeneration (MONET Study). Ophthalmology *119*, 1867–1873.

34. Askou, A.L., Aagaard, L., Kostic, C., Arsenijevic, Y., Hollensen, A.K., Bek, T., Jensen, T.G., Mikkelsen, J.G., and Corydon, T.J. (2015). Multigenic lentiviral vectors for combined and tissue-specific expression of miRNA- and protein-based antiangiogenic factors. Mol. Ther. Methods Clin. Dev. *2*, 14064.

35. Askou, A.L., and Corydon, T.J. (2018). Development of Multigenic Lentiviral Vectors for Cell-Specific Expression of Antiangiogenic miRNAs and Protein Factors. Methods Mol. Biol. *1715*, 47–60.

36. Askou, A.L., Benckendorff, J.N.E., Holmgaard, A., Storm, T., Aagaard, L., Bek, T., Mikkelsen, J.G., and Corydon, T.J. (2017). Suppression of Choroidal Neovascularization in Mice by Subretinal Delivery of Multigenic Lentiviral Vectors Encoding Anti-Angiogenic MicroRNAs. Hum. Gene Ther. Methods *28*, 222–233.

37. Tombran-Tink, J., Aparicio, S., Xu, X., Tink, A.R., Lara, N., Sawant, S., Barnstable, C.J., and Zhang, S.S. (2005). PEDF and the serpins: phylogeny, sequence conservation, and functional domains. J. Struct. Biol. *151*, 130–150.

38. Dawson, D.W., Volpert, O.V., Gillis, P., Crawford, S.E., Xu, H., Benedict, W., and Bouck, N.P. (1999). Pigment epithelium-derived factor: a potent inhibitor of angiogenesis. Science *285*, 245–248.

39. Tombran-Tink, J., Chader, G.G., and Johnson, L.V. (1991). PEDF: a pigment epithelium-derived factor with potent neuronal differentiative activity. Exp. Eye Res. *53*, 411–414.

40. Wang, J.J., Zhang, S.X., Mott, R., Chen, Y., Knapp, R.R., Cao, W., and Ma, J.X. (2008). Anti-inflammatory effects of pigment epithelium-derived factor in diabetic nephropathy. Am. J. Physiol. Renal Physiol. *294*, F1166–F1173.

41. Zhang, S.X., Wang, J.J., Dashti, A., Wilson, K., Zou, M.H., Szweda, L., Ma, J.X., and Lyons, T.J. (2008). Pigment epithelium-derived factor mitigates inflammation and oxidative stress in retinal pericytes exposed to oxidized low-density lipoprotein. J. Mol. Endocrinol. *41*, 135–143.

42. He, X., Cheng, R., Benyajati, S., and Ma, J.X. (2015). PEDF and its roles in physiological and pathological conditions: implication in diabetic and hypoxia-induced angiogenic diseases. Clin. Sci. (Lond.) *128*, 805–823.

43. Boehm, B.O., Lang, G., Volpert, O., Jehle, P.M., Kurkhaus, A., Rosinger, S., Lang, G.K., and Bouck, N. (2003). Low content of the natural ocular anti-angiogenic agent pigment epithelium-derived factor (PEDF) in aqueous humor predicts progression of diabetic retinopathy. Diabetologia *46*, 394–400.

44. Ogata, N., Nishikawa, M., Nishimura, T., Mitsuma, Y., and Matsumura, M. (2002). Unbalanced vitreous levels of pigment epithelium-derived factor and vascular endothelial growth factor in diabetic retinopathy. Am. J. Ophthalmol. *134*, 348–353.

45. Holekamp, N.M., Bouck, N., and Volpert, O. (2002). Pigment epithelium-derived factor is deficient in the vitreous of patients with choroidal neovascularization due to age-related macular degeneration. Am. J. Ophthalmol. *134*, 220–227.

46. Esumi, N., Oshima, Y., Li, Y., Campochiaro, P.A., and Zack, D.J. (2004). Analysis of the VMD2 promoter and implication of E-box binding factors in its regulation. J. Biol. Chem. *279*, 19064–19073.

47. Bennett, J., Pakola, S., Zeng, Y., and Maguire, A. (1996). Humoral response after administration of E1-deleted adenoviruses: immune privilege of the subretinal space. Hum. Gene Ther. *7*, 1763–1769.

48. Lambert, V., Lecomte, J., Hansen, S., Blacher, S., Gonzalez, M.L., Struman, I., Sounni, N.E., Rozet, E., de Tullio, P., Foidart, J.M., et al. (2013). Laser-induced choroidal neovascularization model to study age-related macular degeneration in mice. Nat. Protoc. *8*, 2197–2211.

49. Tong, J.P., and Yao, Y.F. (2006). Contribution of VEGF and PEDF to choroidal angiogenesis: a need for balanced expressions. Clin. Biochem. *39*, 267–276.

50. Hangai, M., Murata, T., Miyawaki, N., Spee, C., Lim, J.I., He, S., Hinton, D.R., and Ryan, S.J. (2001). Angiopoietin-1 upregulation by vascular endothelial growth factor in human retinal pigment epithelial cells. Invest. Ophthalmol. Vis. Sci. *42*, 1617–1625.

51. Kvanta, A. (2006). Ocular angiogenesis: the role of growth factors. Acta Ophthalmol. Scand. *84*, 282–288.

52. Zhang, S.X., Wang, J.J., Gao, G., Parke, K., and Ma, J.X. (2006). Pigment epithelium-derived factor downregulates vascular endothelial growth factor (VEGF) expression and inhibits VEGF-VEGF receptor 2 binding in diabetic retinopathy. J. Mol. Endocrinol. *37*, 1–12.

53. Yancopoulos, G.D., Davis, S., Gale, N.W., Rudge, J.S., Wiegand, S.J., and Holash, J. (2000). Vascular-specific growth factors and blood vessel formation. Nature *407*, 242–248.

54. Zadeh, G., Koushan, K., Pillo, L., Shannon, P., and Guha, A. (2004). Role of Ang1 and its interaction with VEGF-A in astrocytomas. J. Neuropathol. Exp. Neurol. *63*, 978–989.

55. Allocca, M., Doria, M., Petrillo, M., Colella, P., Garcia-Hoyos, M., Gibbs, D., Kim, S.R., Maguire, A., Rex, T.S., Di Vicino, U., et al. (2008). Serotype-dependent packaging of large genes in adeno-associated viral vectors results in effective gene delivery in mice. J. Clin. Invest. *118*, 1955–1964.

56. Dong, B., Nakai, H., and Xiao, W. (2010). Characterization of genome integrity for oversized recombinant AAV vector. Mol. Ther. *18*, 87–92.

57. Lai, Y., Yue, Y., and Duan, D. (2010). Evidence for the failure of adeno-associated virus serotype 5 to package a viral genome > or = 8.2 kb. Mol. Ther. *18*, 75–79.

58. Hirsch, M.L., Agbandje-McKenna, M., and Samulski, R.J. (2010). Little vector, big gene transduction: fragmented genome reassembly of adeno-associated virus. Mol. Ther. *18*, 6–8.

59. Hirsch, M.L., Li, C., Bellon, I., Yin, C., Chavala, S., Pryadkina, M., Richard, I., and Samulski, R.J. (2013). Oversized AAV transductifon is mediated via a DNA-PKcs-independent, Rad51C-dependent repair pathway. Mol. Ther. *21*, 2205–2216.

60. Dougherty, C.J., Smith, G.W., Dorey, C.K., Prentice, H.M., Webster, K.A., and Blanks, J.C. (2008). Robust hypoxia-selective regulation of a retinal pigment epithelium-specific adeno-associated virus vector. Mol. Vis. *14*, 471–480.

61. Aagaard, L.A., Zhang, J., von Eije, K.J., Li, H., Saetrom, P., Amarzguioui, M., and Rossi, J.J. (2008). Engineering and optimization of the miR-106b cluster for ectopic expression of multiplexed anti-HIV RNAs. Gene Ther. *15*, 1536–1549.

62. Corydon, T.J., Haagerup, A., Jensen, T.G., Binderup, H.G., Petersen, M.S., Kaltoft, K., Vestbo, J., Kruse, T.A., and Børglum, A.D. (2007). A functional CD86 polymorphism associated with asthma and related allergic disorders. J. Med. Genet. *44*, 509–515.

63. Bemelmans, A.P., Kostic, C., Crippa, S.V., Hauswirth, W.W., Lem, J., Munier, F.L., Seeliger, M.W., Wenzel, A., and Arsenijevic, Y. (2006). Lentiviral gene transfer of RPE65 rescues survival and function of cones in a mouse model of Leber congenital amaurosis. PLoS Med. *3*, e347.

64. Gong, Y., Li, J., Sun, Y., Fu, Z., Liu, C.H., Evans, L., Tian, K., Saba, N., Fredrick, T., Morss, P., et al. (2015). Optimization of an Image-Guided Laser-Induced Choroidal Neovascularization Model in Mice. PLoS ONE *10*, e0132643.

65. Holmgaard, A., Askou, A.L., Benckendorff, J.N.E., Thomsen, E.A., Cai, Y., Bek, T., Mikkelsen, J.G., and Corydon, T.J. (2017). In Vivo Knockout of the Vegfa Gene by Lentiviral Delivery of CRISPR/Cas9 in Mouse Retinal Pigment Epithelium Cells. Mol. Ther. Nucleic Acids *9*, 89–99.

66. Winer, J., Jung, C.K., Shackel, I., and Williams, P.M. (1999). Development and validation of real-time quantitative reverse transcriptase-polymerase chain reaction for monitoring gene expression in cardiac myocytes in vitro. Anal. Biochem. *270*, 41–49.

OMTN, Volume *16*

Supplemental Information

Suppression of Choroidal Neovascularization by AAV-Based Dual-Acting Antiangiogenic Gene Therapy

Anne Louise Askou, Sidsel Alsing, Josephine N.E. Benckendorff, Andreas Holmgaard, Jacob Giehm Mikkelsen, Lars Aagaard, Toke Bek, and Thomas J. Corydon

Figure S1

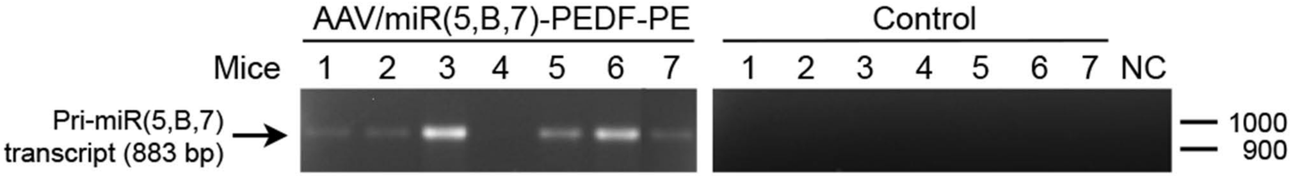
AAV/miR(5,B,7)-PEDF-PE
Control
Mice 1 2 3 4 5 6 7
1 2 3 4 5 6 7 NC
Pri-miR(5,B,7)
transcript (883 bp)
1000
900

Figure S2

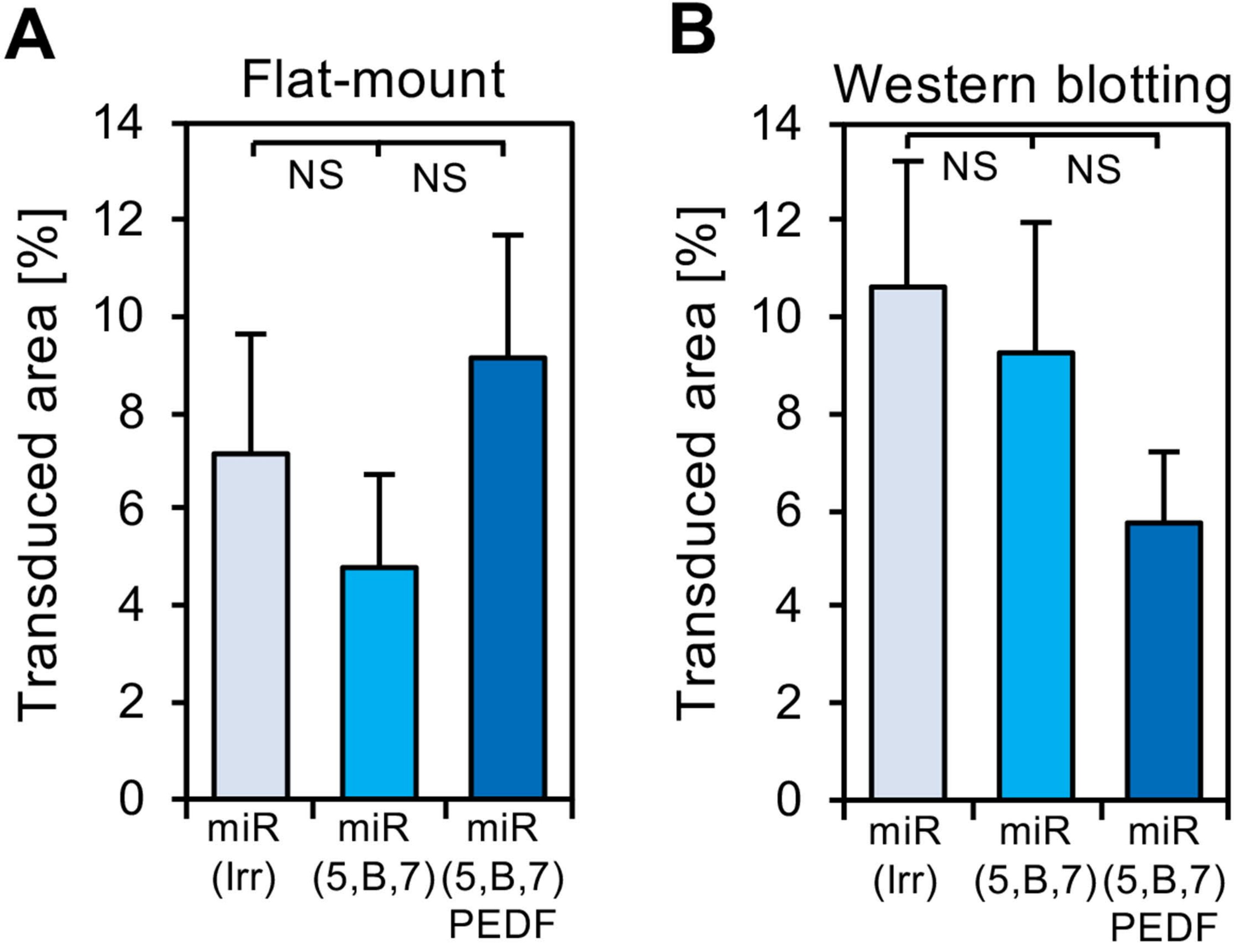

A
Flat-mount
Transduced area [%]
14
12
10
8
6
4
2
0
NS
NS
miR
(Irr)
miR
(5,B,7)
miR
(5,B,7)
PEDF
B
Western blotting
Transduced area [%]
14
12
10
8
6
4
2
0
NS
NS
miR
(Irr)
miR
(5,B,7)
miR
(5,B,7)
PEDF

Figure S3

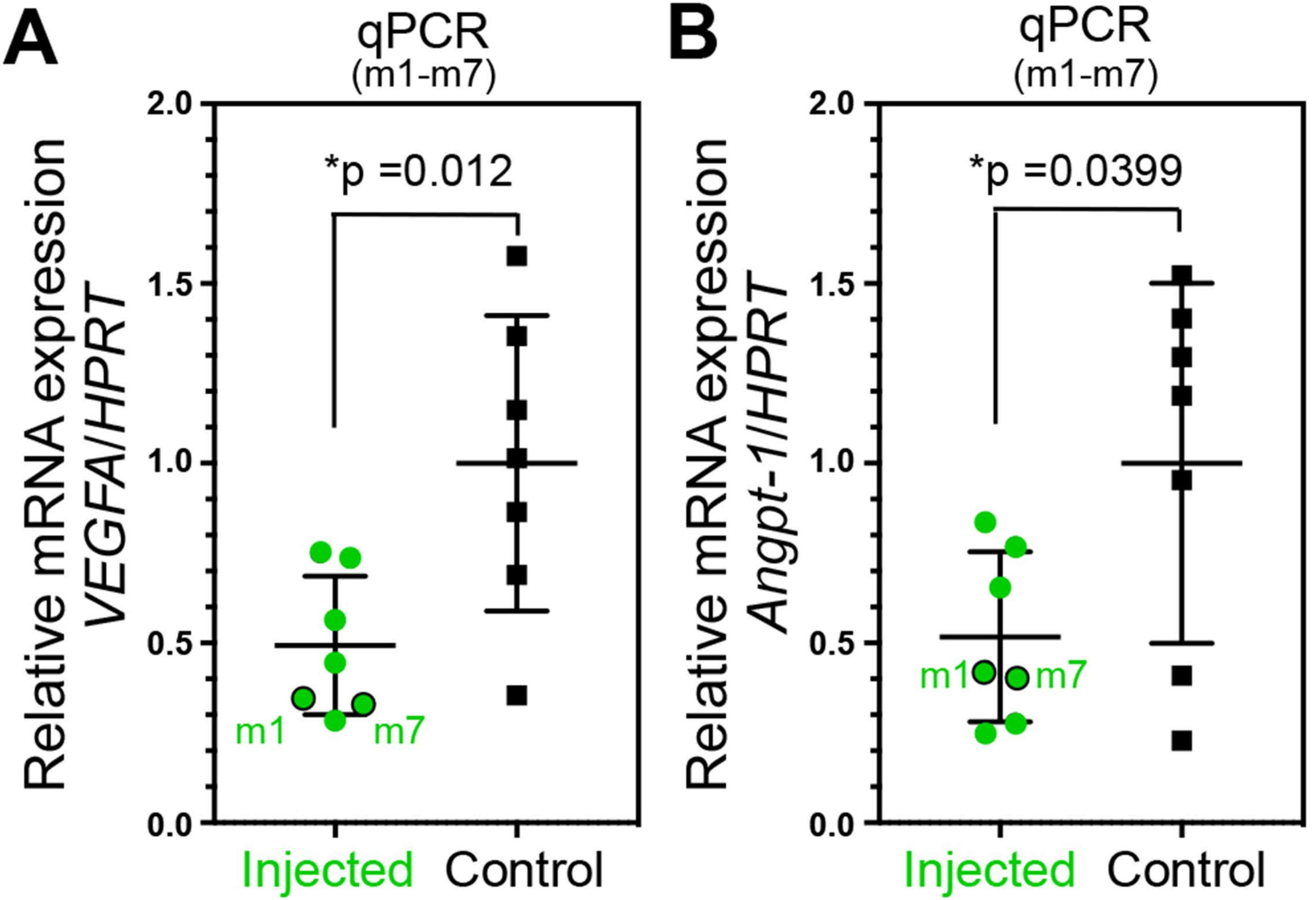
A
qPCR
(m1-m7)
Relative mRNA expression
VEGFA/HPRT
2.0
1.5
1.0
0.5
0.0
*p =0.012
m1
m7
Injected
Control
B
qPCR
(m1-m7)
Relative mRNA expression
Angpt-1/HPRT
2.0
1.5
1.0
0.5
0.0
*p =0.0399
m1
m7
Injected
Control

Figure S4

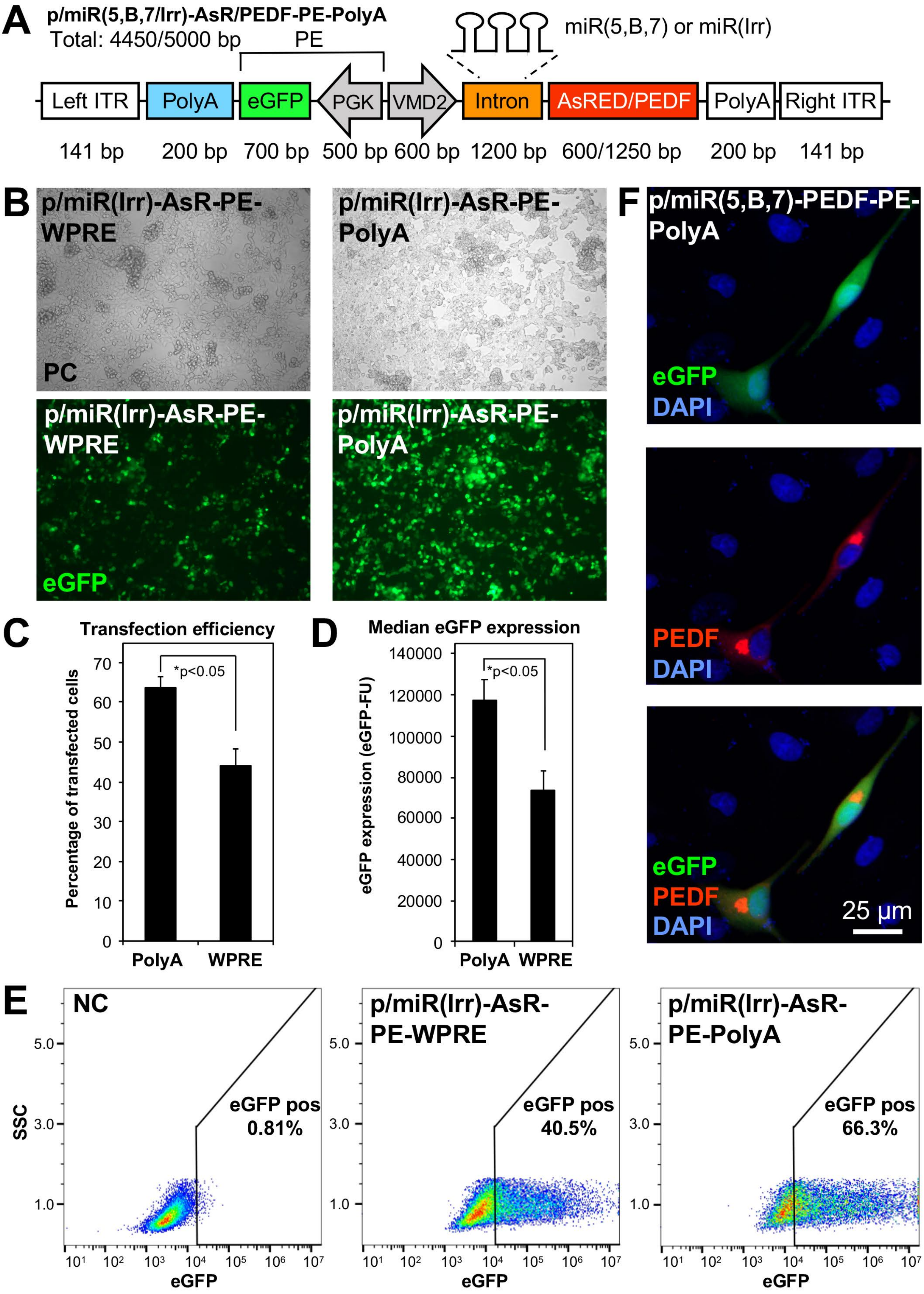
A
p/miR(5,B,7/Irr)-AsR/PEDF-PE-PolyA
Total: 4450/5000 bp
PE
miR(5,B,7) or miR(Irr)
Left ITR
PolyA
eGFP
PGK
VMD2
Intron
AsRED/PEDF
PolyA
Right ITR
141 bp
200 bp
700 bp
500 bp
600 bp
1200 bp
600/1250 bp
200 bp
141 bp
B
p/miR(Irr)-AsR-PE-WPRE
p/miR(Irr)-AsR-PE-PolyA
PC
p/miR(Irr)-AsR-PE-WPRE
p/miR(Irr)-AsR-PE-PolyA
eGFP
C
Transfection efficiency
Percentage of transfected cells
*p<0.05
PolyA
WPRE
D
Median eGFP expression
eGFP expression (eGFP-FU)
*p<0.05
PolyA
WPRE
E
NC
p/miR(Irr)-AsR-PE-WPRE
p/miR(Irr)-AsR-PE-PolyA
SSC
eGFP pos 0.81%
eGFP pos 40.5%
eGFP pos 66.3%
eGFP
F
p/miR(5,B,7)-PEDF-PE-PolyA
eGFP
DAPI
PEDF
DAPI
eGFP
PEDF
DAPI
25 µm

Figure S1 Expression of miRNA precursor transcript pri-miR(5,B,7) from AAV/miR(5,B,7)-PEDF-PE in the retina. Expression of the pri-miR(5,B,7) transcript from AAV/miR(5,B,7)-PEDF-PE in the retina was examined by RT-PCR. Twenty-eight dpi RPE cells were isolated from enucleated eyes and analyzed by PCR using cDNA as described in Materials and Methods. The contralateral, uninjected eye was used as control. A sample without cDNA was used as negative control (NC). Position of the pri-miR(5,B,7) transcript with the predicted size of 883 bp is indicated to the left. Positions of the 1000 and 900 bp bands of the molecular ruler are shown to the right. Bp, base pairs; NC, negative control.

Figure S2 Quantification of eGFP expression in the retina following delivery of AAV/miR(Irr/5,B,7)-AsRED 50 dpi. eGFP expression was assessed by fundoscopy in injected eyes included in the analysis shown in Figure 5 (n = 56). **A** Transduced area (percentage of total analyzed area) in the flat-mount group (n = 28). **B** Transduced area (percentage of total analyzed area) in the Western blotting group (n = 28). No statistically significant (indicted by NS) difference between the three groups was observed (one-way ANOVA followed by multiple comparisons (Tukey's multiple comparison test)), p-values in the range of 0.37-0.82 (flat-mount), and 0.34-0.92 (Western blotting). Error bars indicate SEM. Dpi, day post-injection; miR, microRNA; PEDF, pigment epithelium-derived factor.

Figure S3 Quantification of *VEGFA* and *Angpt-1* expression in RPE cells following subretinal delivery of AAV/miR(5,B,7)-PEDF-PE. In order to investigate the effect of reduced *VEGFA* levels on the angiogenic pathway *in vivo*, mRNA levels of the target *VEGFA* and of *Angpt-1* were assessed in RPE cells following a single subretinal injection (m1-m7). Twenty-eight dpi RPE cells were isolated from enucleated eyes and analyzed by RT-qPCR as described in Materials and Methods. Uninjected eyes were used as negative control. Mouse 1 and 7 indicated (green dots with black lines). **A** Assessment of the *VEGFA* expression (p = 0.012). **B** Assessment of the *Angpt-1* expression (p = 0.0399). The relative expression of *VEGFA* and *Angpt-1* in uninjected eyes were set to 1. *Statistically significant. Error bars indicate SEM. Angpt-1, Angiopoietin-1; dpi, day post-injection; miR, microRNA; PE, PGK-eGFP; PEDF, pigment epithelium-derived factor; VEGFA, Vascular endothelial growth factor A.

Figure S4 Optimization of the multigenic AAV vector. A Schematic presentation of the optimized p/miR(5,B,7/Irr)-AsR/PEDF-PE-PolyA vector. The WPRE sequence of the original vector presented in Figure 1 was replaced with the PolyA sequence. Numbers indicate size (in bp of the various components). The total sizes (in kb) of p/miR(5,B,7)-AsR/PEDF-PE-PolyA and p/miR(Irr)-AsR/PEDF-PE-PolyA are likewise indicated. **B** Visualization of eGFP expression in HEK-293 cells transfected with either p/miR(Irr)-AsR-PE-WPRE or p/miR(Irr)-AsR-PE-PolyA. Original magnification x10. **C-E** Flow cytometry analysis of HEK-293 cells transfected with either p/miR(Irr)-AsR-PE-WPRE or p/miR(Irr)-AsR-PE-PolyA. Untransfected cells were used as NC. **C-D** Assessment of the transfection efficiency and median eGFP expression following flow cytometry (mean ± SD). Students t-test, p = 0.006 (transfection efficiency) and p = 0.011 (expression). *Statistically significant. **E** Flow cytometry diagrams used to calculate the findings presented in C-D. The gaiting of the eGFP-positive cells is indicated. **F** Expression of eGFP (green) and PEDF in melanoma cells following transfection with p/miR(5,B,7)-PEDF-PE-PolyA. Visualization of PEDF was obtained following immunostaining using a mouse anti-PEDF-antibody. For visualization anti-PEDF antibodies were labeled with 568-ALEXA-anti-mouse antibodies (red). To visualize nuclei cells were stained with DAPI (blue). Scale bar = 25 µm. AsRED/AsR, red fluorescent marker protein; bp, base pairs; DAPI, 4',6-diamidino-2-phenylindol; eGFP, enhanced green fluorescent protein; FU, fluorescent unit; HEK-293, human embryonic kidney cells; H3, histone H3; Irr, irrelevant; ITR, inverted terminal repeat; kb, kilo bases; miR, microRNA; NC, negative control (untransfected cells); PE, PGK-eGFP; PEDF, pigment epithelium-derived factor; PC, phase contrast; PGK, phosphoglycerate kinase 1 promoter; PolyA, polyadenylation signal; SSC, side scatter; VMD2, vitelliform macular dystrophy 2 promoter.
